# Synthesis, dynamic NMR characterization and XRD studies of novel *N*,*N*’-substituted piperazines for bioorthogonal labeling

**DOI:** 10.3762/bjoc.12.242

**Published:** 2016-11-21

**Authors:** Constantin Mamat, Marc Pretze, Matthew Gott, Martin Köckerling

**Affiliations:** 1 Helmholtz-Zentrum Dresden-Rossendorf, Institut für Radiopharmazeutische Krebsforschung, Bautzner Landstraße 400, D-01328 Dresden, Germany; 2 Technische Universität Dresden, Fachrichtung Chemie und Lebensmittelchemie, D-01062 Dresden, Germany; 3 Medizinische Fakultät Mannheim der Universität Heidelberg, Institut für Klinische Radiologie und Nuklearmedizin, Theodor-Kutzner-Ufer 1-3, D-68167 Mannheim, Germany; 4 Universität Rostock, Institut für Chemie – Festkörperchemie, Albert-Einstein-Straße 3a, D-18059 Rostock, Germany

**Keywords:** building blocks, coalescence, dynamic NMR, labeling, Staudinger ligation

## Abstract

Novel, functionalized piperazine derivatives were successfully synthesized and fully characterized by ^1^H/^13^C/^19^F NMR, MS, elemental analysis and lipophilicity. All piperazine compounds occur as conformers resulting from the partial amide double bond. Furthermore, a second conformational shape was observed for all nitro derivatives due to the limited change of the piperazine chair conformation. Therefore, two coalescence points were determined and their resulting activation energy barriers were calculated using ^1^H NMR. To support this result, single crystals of 1-(4-nitrobenzoyl)piperazine (**3a**, monoclinic, space group *C*2/*c*, *a* = 24.587(2), *b* = 7.0726(6), *c* = 14.171(1) Å, β = 119.257(8)°, *V* = 2149.9(4) Å^3^, *Z* = 4, *D*_obs_ = 1.454 g/cm^3^) and the alkyne derivative 4-(but-3-yn-1-yl)-1-(4-fluorobenzoyl)piperazine (**4b**, monoclinic, space group *P*2_1_/*n*, *a* = 10.5982(2), *b* = 8.4705(1), *c* = 14.8929(3) Å, β = 97.430(1)°, *V* = 1325.74(4) Å^3^, *Z* = 4, *D*_obs_ = 1.304 g/cm^3^) were obtained from a saturated ethyl acetate solution. The rotational conformation of these compounds was also verified by XRD. As proof of concept for future labeling purposes, both nitropiperazines were reacted with [^18^F]F^–^. To test the applicability of these compounds as possible ^18^F-building blocks, two biomolecules were modified and chosen for conjugation either using the Huisgen-click reaction or the traceless Staudinger ligation.

## Introduction

The development of new building blocks for specific and bioorthogonal labeling of biologically active compounds is of high importance. Depending on their size and composition, building blocks can influence (bio-)chemical parameters such as the lipophilicity (log *P*) affecting the solubility and the biological behavior of the resulting labeled biomolecule [1]. To diversify and regulate this behavior, compounds with a piperazine skeleton were chosen as excellent candidates for bispecific modification.

Simple alkylated and acylated secondary amines/amides are known from the literature [2] as well as their NMR properties [3]. The widely used solvent DMF is an intensely investigated example [4]. Functionalized piperazines [5], especially benzoylated derivatives, are our interest. In the past, mono or bisacylated piperazines with benzamide [6], nicotinamide [7] and isonicotinamide [8–11] residue were explored in terms of their NMR and complexation behavior. However, unsymmetrically substituted piperazines are rarely investigated [12–13].

*N*,*N*’-Unsymmetrically functionalized piperazines are the basis of the development of our new building blocks. In this case, one of the nitrogen atoms is used for the connection to the label (e.g., fluorescence dye, radionuclide) and the second is used for the introduction of a (bioorthogonal) functional group (e.g., azide, alkyne, phosphane, tetrazine) to later connect to the biomolecule via bioorthogonal ligation.

Our aim was the development of novel, *N*,*N*’-unsymmetrically functionalized piperazine derivatives using a high-yielding, simple synthesis with either a bioorthogonal alkyne or azide functionality for future labeling purposes. Furthermore, characterization of these piperazine derivatives was performed with emphasis on their particular NMR behavior. In order to demonstrate the Cu-catalyzed azide–alkyne click reaction (Huisgen 1,3-dipolar cycloaddition) and the traceless Staudinger ligation, a proof of concept study was performed for the site-selective labeling of a pharmacologically active peptide and a small organic compound. These compounds provide the respective reference compounds for later radiolabeling purposes. Finally, a procedure to synthesize the building blocks containing fluorine-18 was evaluated.

## Results and Discussion

### Synthesis of the piperazine compounds

**Scheme 1:**
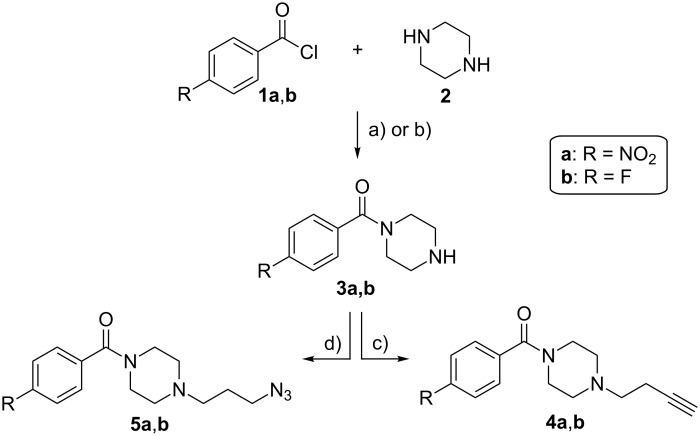
Preparation of the nitro derivatives **4a** and **5a** and the fluorine-containing compounds **4b** and **5b**. Reagents and conditions: a) **1a**, Et_3_N, CHCl_3_, 0 °C, 2 h, 0 °C then 2 h, rt; b) **1b**, acetonitrile, 1 M HCl, rt, 4 h; c) 4-tosylbutyne, Et_3_N, THF, 60 °C, 3 d; d) 3-azidopropyl tosylate, Et_3_N, THF, 60 °C, 3 d.

Low-cost starting materials were applied for the preparation of all novel building blocks. First, piperazine (**2**) was reacted with functionalized benzoyl chlorides **1a**,**b** to yield the mono-acylated amides **3a**,**b** according to literature procedures [14]. Next, the necessary functional groups for the later click reactions to connect the resulting building blocks to biomolecules were introduced. Thus, **3a**,**b** were alkylated with 4-tosylbutyne to give compounds **4a**,**b** in high yields of 84% and 82%, respectively. These compounds are applicable in the classical Cu-catalyzed Huisgen-click reaction with azide-functionalized, biologically active molecules. Additionally, **3a**,**b** were reacted with 3-azidopropyl tosylate to yield compounds **5a**,**b** in high yields of 87% and 81%, respectively. These derivatives can be utilized for both variants of the Staudinger ligation in addition to the classical and strain-promoted variants of the Huisgen-click reaction. For future radiolabeling procedures, nitro derivatives **4a** and **5a** serve as starting material (precursor) whereas fluorine compounds **4b** and **5b** function as appendant reference compounds to analyze the prospective ^18^F-containing compounds. The reaction pathway for all piperazines is illustrated in Scheme 1.

### Dynamic NMR studies

During the full characterization of our compounds, we observed a quite unusual behavior in the ^1^H and ^13^C NMR spectra. Four broad singlets (ratio: 1:1:1:1) were observed in the ^1^H NMR spectra of all nitro compounds **3a**, **4a**, and **5a** (see Supporting Information File 1) and three broad singlets (ratio 1:1:2) were determined for all fluorine compounds **3b**, **4b**, and **5b** measured in CDCl_3_ at 25 °C. Normally, under these conditions only two signals are expected for the NCH_2_-protons of unsymmetrically substituted piperazines [15–17].

**Figure 1:**
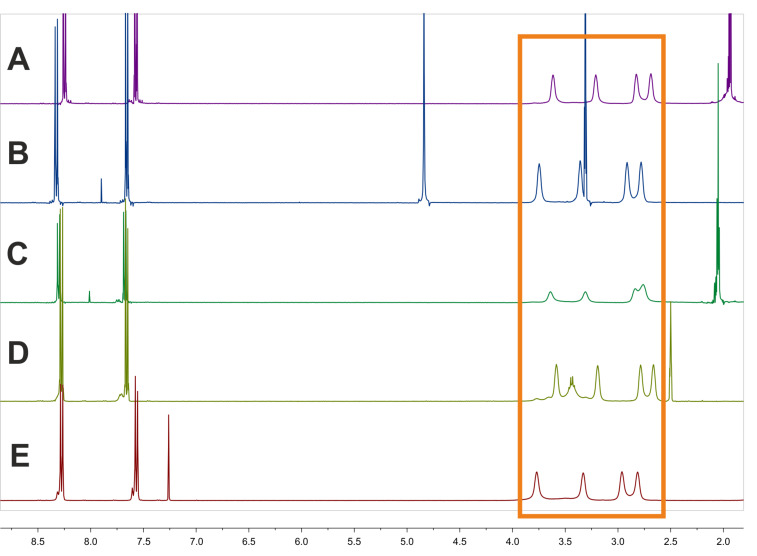
^1^H NMR spectra of compound **3a** measured in five different solvents: (A) CDCl_3_, (B) DMSO-*d*_6_, (C) acetone-*d*_6_, (D) methanol-*d*_4_ and (E) acetonitrile-*d*_3_ (orange: region of interest).

**Figure 2:**
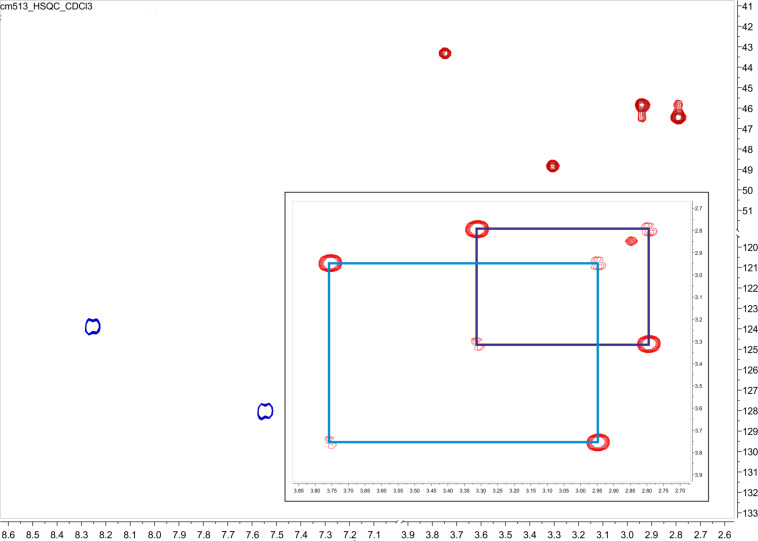
HSQC and H,H-COSY (small spectrum) of compound **3a** measured in CDCl_3_ at 25 °C. The independent coupling of the NCH_2_ groups is marked.

To investigate this phenomenon, nitro compound **3a** was chosen and ^1^H NMR spectra were measured in five different solvents to confirm this behavior. The results are illustrated in Figure 1. As an example, the spectrum of **3a** shows four broad signals (δ = 2.81, 2.96, 3.33, 3.97 ppm in CDCl_3_ at 25 °C) for the piperazine NCH_2_ groups and evaluation of a H,H-COSY measured in CDCl_3_ showed an independent coupling of two NCH_2_ groups (Figure 2). Next, the HSQC spectra showed the independent coupling of the protons to the appropriate carbon signals (further detailed NMR spectra can be found in Supporting Information File 1). Additionally, four broad signals for the carbons of the NCH_2_ groups (e.g., **3a**: δ = 43.7, 46.0, 46.3, 49.0 ppm) are found when analyzing the ^13^C NMR spectra.

**Figure 3:**
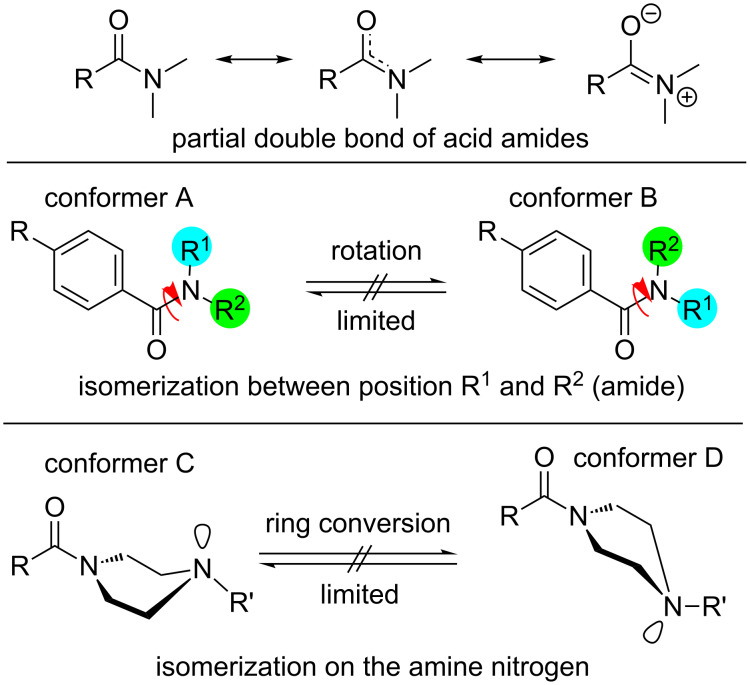
Illustration of the general partial double bond character of an amide bond and the limited isomerization of the amine nitrogen atom which results in possible conformers of piperazine compounds.

Two effects are responsible for this behavior. The first arises from the presence of two different conformers (rotamers); this is caused by the limited interconversion by rotation about the C–N amide bond resulting from the partial double bond character of *N*,*N*-dialkylated amides as shown in Figure 3. This observation is typical and can be found for symmetrically substituted amides. The best known and widest investigated example is DMF. Two distinct signals are observed in the ^1^H NMR spectrum of DMF as a result of two structurally different methyl groups (R^1^ ≠ R^2^) attached to the amide nitrogen [18–19]. In general, this behavior of symmetric *N*,*N*-dialkylamide spin systems is describable as first-order process on the NMR time scale [20].

The same NMR behavior is observed for 4-nitrobenzoyl amides **3a**, **4a** and **5a**. The expected single signal of the CH_2_ groups attached to the amide nitrogen is duplicated as seen for **3a** in Figure 1. The coupling is pointed out by the analysis of a H,H-COSY of **3a** (Figure 2, small spectrum).

The second effect is related to the reduced flipping of the piperazine ring at the amine nitrogen (Figure 3, bottom). In our case, interconversion of the amine is also reduced at room temperature. Normally, such formation of conformers is found for piperazines [21–22] and morpholines [21,23] only at lower temperatures (below −10 °C). Additionally, only the protons of 4-nitrobenzoylamides **3a**, **4a** and **5a** exhibit this behavior at room temperature. Consequently, this phenomenon is strongly influenced by the substituent in the *para*-position of the benzoate (F vs NO_2_).

Both effects resulted in two different coalescence points dependent upon their different energy barriers. In order to further investigate the conformational behavior of the piperazines and to determine these energies, temperature-dependent NMR experiments [24] were performed for all nitro derivatives **3a**, **4a**, and **5a** as well as for fluorine compound **3b**. When monitoring compound **3a** over a minimum range of 45 K, the four signals of the NCH_2_ groups gradually disappear and coalesce to the two expected signals at increased temperatures (>67 °C). At the coalescence temperature *T*_c_, the exchange rate is given by the equation *k*_exc_ = π·Δν/2^1/2^ [25]. As a result, the activation energy (Δ*G*^#^_exp_) to the amide bond rotation can be calculated using the Eyring equation [14,16].


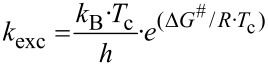


In general, the difference in chemical shifts Δδ (as well as Δν) strongly depends on the nature of the solvent [26]. Thus, the ^1^H NMR spectra seen in Figure 1 were recorded in CDCl_3_, DMSO-*d*_6_, acetone-*d*_6_, methanol-*d*_4_ and acetonitrile-*d*_3_ at 400 MHz and the results are summarized in Table 1. The NCH_2_ groups of **3a** show the highest difference Δν_1_ and Δν_2_ when dissolved in CDCl_3_ and the lowest when dissolved in acetone-*d*_6_.

**Table 1:** NMR parameters Δν and *k*_exc_ of nitro compound **3a** measured in dependence of the solvent.

solvent	Δν_1_ [Hz]	Δν_2_ [Hz]	*k*_exc,1_ [Hz]	*k*_exc,2_ [Hz]

CDCl_3_	59.5	176.2	132.2	391.4
acetonitrile-*d*_3_	55.2	162.2	122.6	360.3
DMSO-*d*_6_	49.5	157.6	110.0	350.1
methanol-*d*_4_	53.3	155.8	118.4	346.1
acetone-*d*_6_	30.4	133.2	67.5	295.9

**Figure 4:**
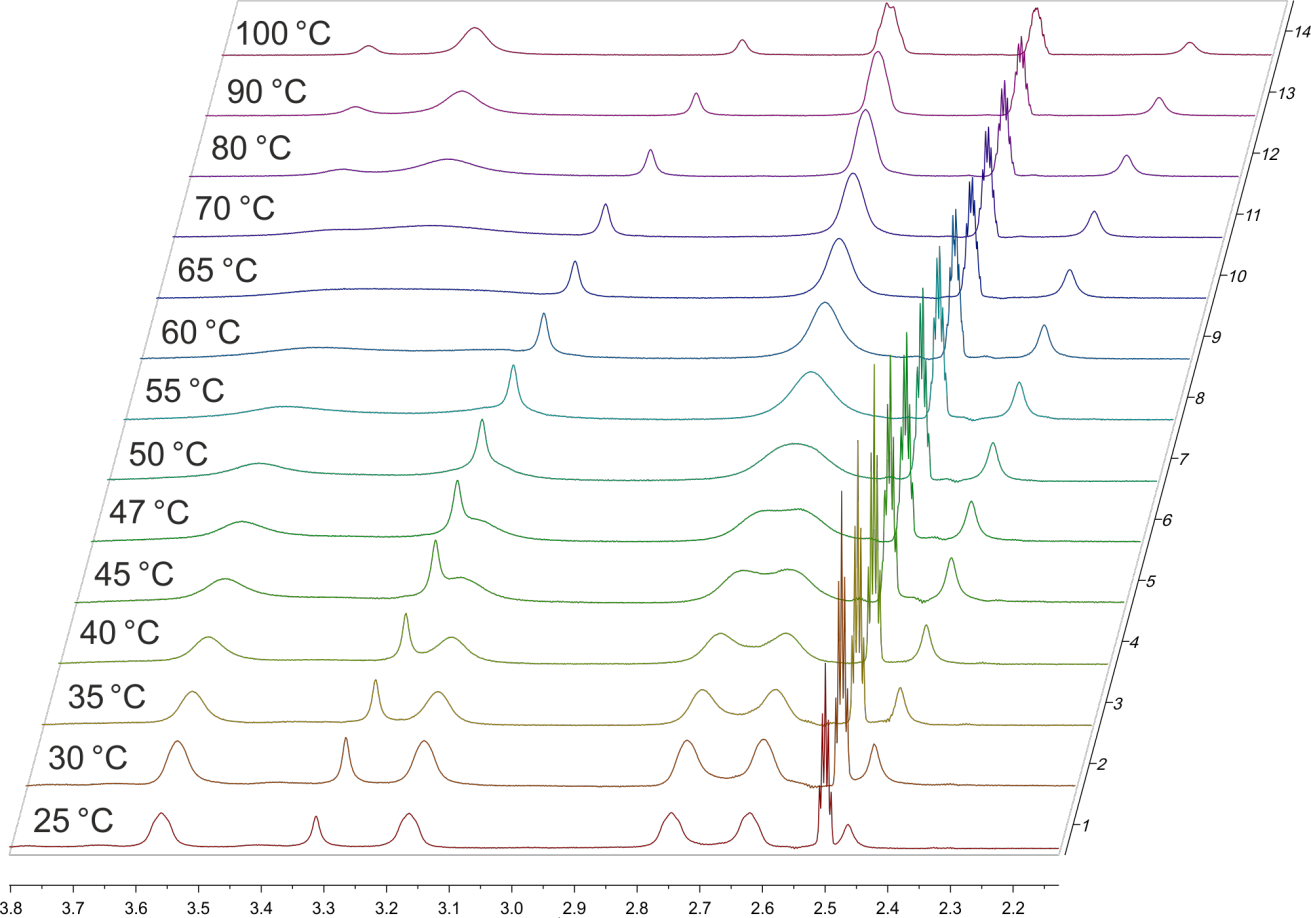
Temperature-dependent ^1^H NMR spectra of **3a** measured in DMSO-*d*_6_ (aliphatic region of the piperazine protons from 2.00 to 4.00 ppm is shown).

Due to the low boiling point of most of these solvents, DMSO-*d*_6_ was chosen for the measurement of the coalescence temperature via temperature-dependent ^1^H NMR (Figure 4). For **3a**, the *T*_c,1_ was determined to be 50 °C (322 K) and *T*_c,2_ was 67 °C (340 K). Using this data, the activation energies Δ*G*^#^_exc_ were calculated to be 66.7 and 67.1 kJ/mol, respectively (Table 2).

**Table 2:** Evaluation of the Gibb’s energy Δ*G*^#^_exc_ of compounds **3a**, **3b**, **4a** and **5a** from NMR data measured in DMSO-*d*_6_.

	Δν_1_	Δν_2_	*k*_exc,1_	*k*_exc,2_	*T*_c,1_ [K]	*T*_c,2_ [K]	Δ*G*^#^_exc,1_ [kJ/mol]	Δ*G*^#^_exc,2_ [kJ/mol]

**3a**	49.5	157.6	110.0	350.1	323	340	66.7	67.1
**3b**	n.d.	106.6	n.d.	236.8	n.d.	306	n.d.	61.1
**4a**	48.5	154,0	107.7	342.1	320	338	66.1	66.7
**5a**	48.6	154.9	108.0	344.1	315	338	65.0	66.7

For fluorine compound **3b**, only the CH_2_ group attached to the amide nitrogen is split in two signals at room temperature and its *T*_c_ was found to be 33 °C (306 K) resulting in a Δ*G*^#^_exc_ of 61.1 kJ/mol. This result nicely demonstrates the influence of the substituent in the ortho-position (F vs NO_2_) of the benzoate residue.

Next, the influence of alkyl groups on *T*_c_ and Δ*G*^#^_exp_ were investigated using both nitrobenzoylpiperazines **4a** and **5a**. Both coalescence temperatures are found to be lower than those of the non-alkylated derivative **3a**. This effect could arise due to the increased steric demand of the alkyl groups compared to the sole hydrogen connected to the amine nitrogen.

When comparing our results with the literature, *T*_c_ and Δ*G*^#^_exp_ found for the amide site of the piperazines are in good agreement with the previously published (*T*_c_ = 330–340 K, Δ*G*^#^_exp_ = 61–68 kJ/mol) [21–22,27]. In contrast, our values for *T*_c,1_ and Δ*G*^#^_exp,1_ for the amine residues are much higher as found in the literature. For instance, *N*-alkylated morpholines showed a *T*_c_ of 248 K with a Δ*G*^#^ of 11.1 kJ/mol [23] whereas piperidine shows a higher Δ*G*^#^ of 42.3 and *N*-methylpiperidine a Δ*G*^#^ = 49.8 kJ/mol [21,28].

### X-ray structure analyses of 3a and 4b

**Figure 5:**
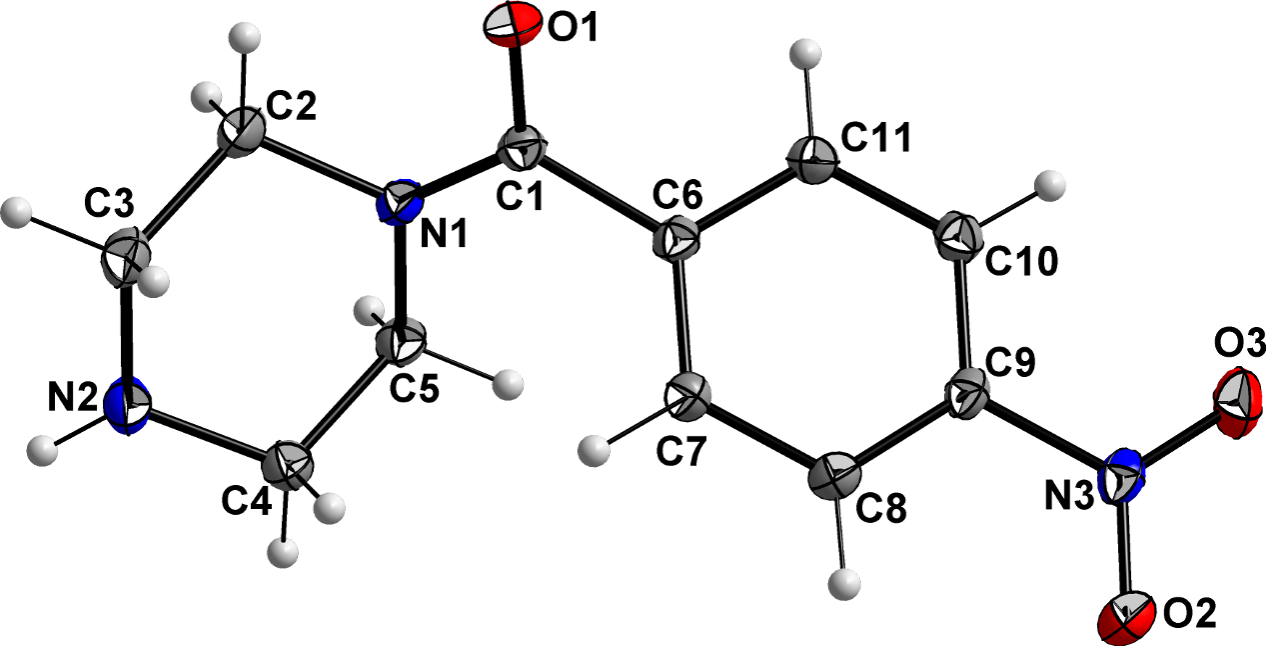
Molecular structure of compound **3a** (ORTEP plot with 50% probability level).

**Figure 6:**
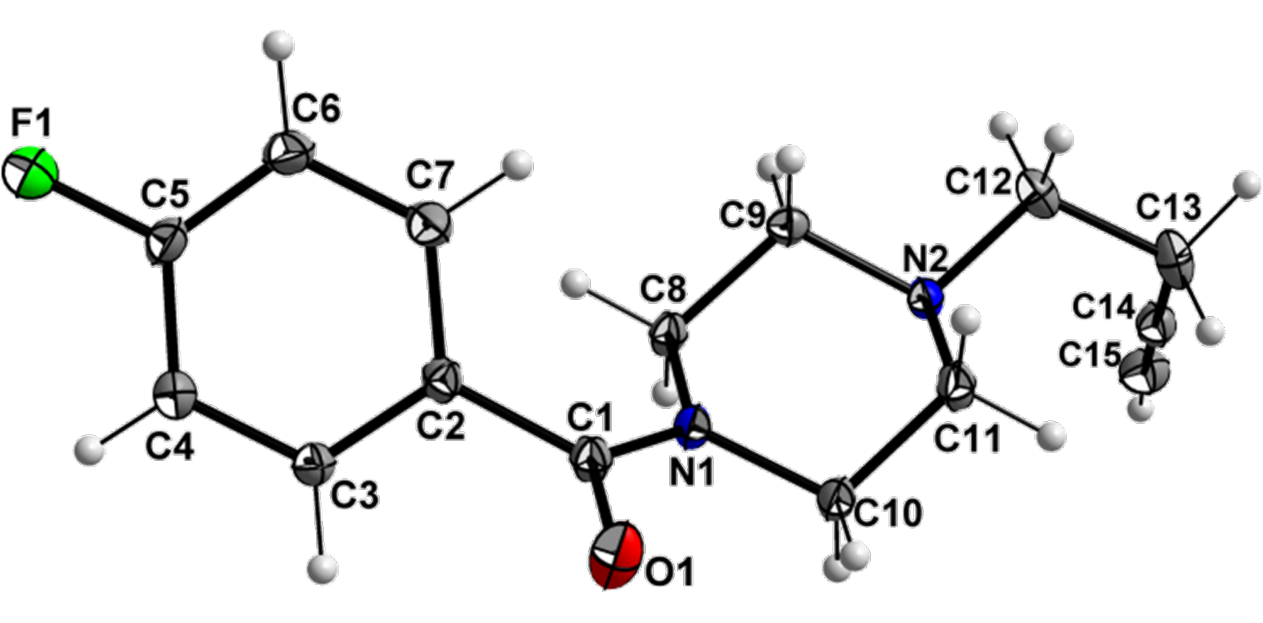
Molecular structure of compound **4b** (ORTEP plot with 50% probability level).

Single crystals of **3a** and **4b** were obtained and their molecular structures determined using single crystal X-ray structure analysis. Crystals of **3a** have monoclinic symmetry of the space group *C*2/*c*. Crystals of **4b** have monoclinic symmetry of the space group *P*2_1_/*n*. The C14–C15 distance of 1.188(1) Å and the C13–C14–C15 angle of 178.2(1)° clearly indicate this group to be an alkyne residue, thus enabling the use of the Huisgen-click reaction for binding to the target biomacromolecule. The molecular structures of these compounds and their atom numbering schemes are shown in Figure 5 and Figure 6.

The limited ability of the residues to rotate along the C1–N1 bond, as observed by NMR spectroscopy (see above), is supported by the results of both single-crystal structure determinations. The amine-type N2 atom in **4b** has three bonds to the neighboring carbon atoms of about the same length (N2–C9: 1.466(1) Å, N2–C11: 1.466(1) Å, N2–C12: 1.464(1) Å), but the amide-type N1 atom has a significantly shorter distance to the C1 atom (1.350(1) Å), which carries the carbonyl O atom. The other two contacts of N1 to the C atoms of the piperazine ring are in the same distance range (N1–C8: 1.461(1) Å and N1–C10: 1.461(1) Å) as those of the N2 atom.

**Figure 7:**
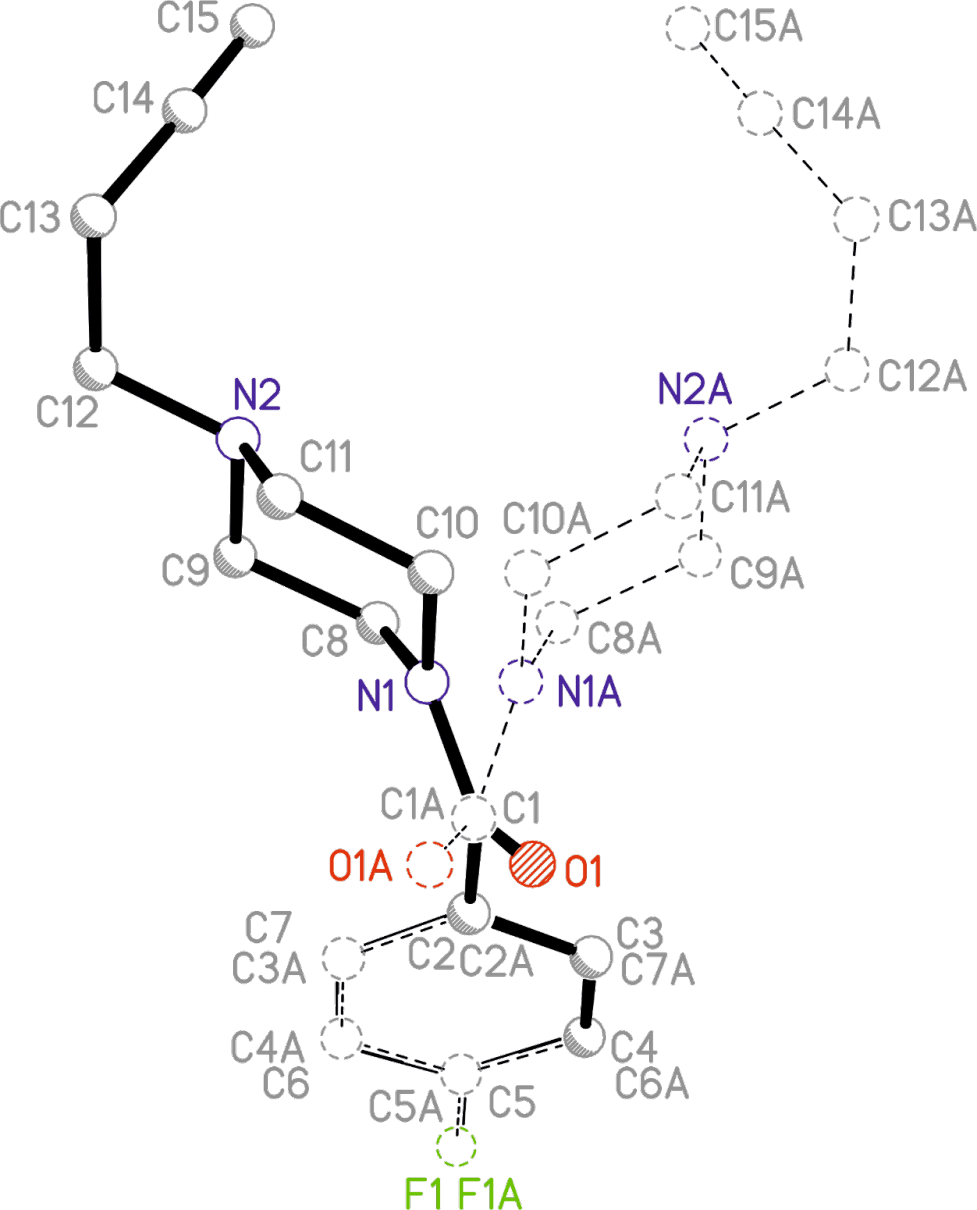
Superimposition fit of the two conformers, which exist in the ratio of 1:1 in the solid state structure of compound **4b** (label A marks the atoms of the second conformer).

Furthermore, the environment of the N2 atom can best described as pyramidal with an average C–N2–C angle of 109.8°, whereas the environment of the N1 atom is almost planar, with an average C–N1–C angle of 119.9°. All these results indicate a partial double-bond character for the C1–N1 bond with a limited rotational ability. As discussed above, this results in two conformers, as shown for solutions of **4b** by NMR spectroscopy. In the solid state, the molecules of the asymmetric unit are located on a side without any symmetry (besides identity). Through the symmetry element besides the molecule (i.e., inversion center) in the solid state, two conformers exist in a ratio of 1:1. Figure 7 shows a superimposed figure of the two conformers, where C1, the phenyl ring and F1 of both conformers are fitted on top of each other. The different structural arrangement of both conformers is clearly visible.

Similar structural features are observed for **3a**. The N1–C1 atom distance is again much shorter, 1.346(2) Å, than the other N–C bonds within the piperazine moiety, which range from 1.462(2) to 1.466(1) Å. Furthermore, the average bond angle of 119.8° of N1 indicates double-bond character and thereby limited rotational ability of the residues along the N1–C1 bond. As found for **4b**, crystals of **3a** contain two isomers in a 1:1 ratio as imposed by crystal symmetry.

### Sample ligation using Huisgen-click and traceless Staudinger

**Scheme 2:**
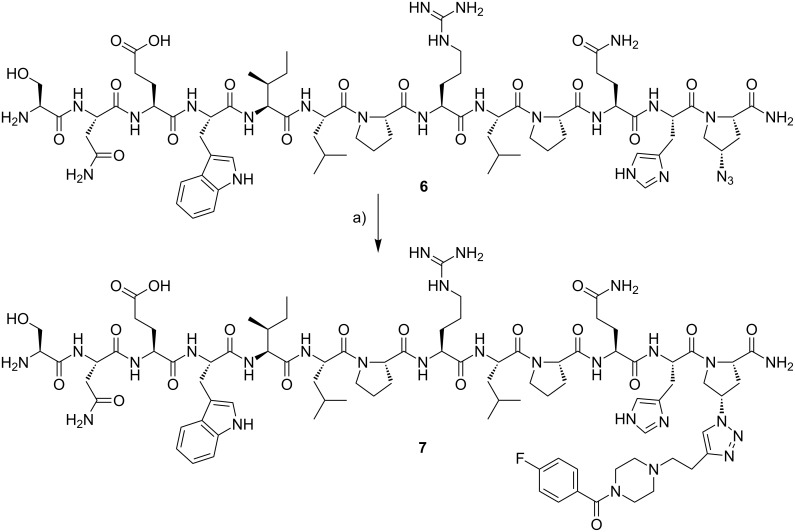
Peptide labeling using the Huisgen-click reaction and building block **4b**. Reagents and conditions: a) **4b**, CuSO_4_, Na ascorbate, PBS buffer (pH 7.4), 40 °C, 16 h.

As a proof of labeling concept, fluorine compound **4b** was clicked to peptide **6** using the Cu-catalyzed Huisgen-click reaction. This SNEW peptide (SNEW: Ser-Asn-Glu-Trp) was chosen due to its biologically and pharmacologically activity and was modified with an azide moiety at the C-terminus to yield SNEWILPRLPQH-Azp **6** (the synthesis is reported elsewhere) [29]. The structure of **6** and the click reaction is shown in Scheme 2. For labeling purposes, building block **4b** (10-fold excess) was added to peptide **6** which was dissolved in PBS buffer (pH 7.4), followed by addition of freshly prepared solutions of Na ascorbate and CuSO_4_. The mixture was stirred for 16 h at 40 °C and the desired product **7** was purified by semi-preparative HPLC.

**Scheme 3:**
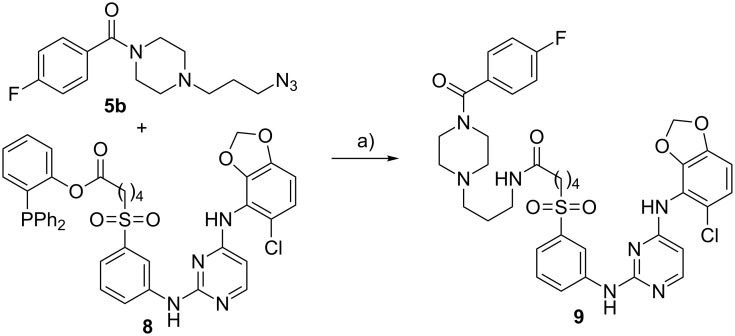
The traceless Staudinger ligation to yield compound **9**. Reagents and conditions: a) acetonitrile/water 10:1 (v/v), 60 °C, 3 h.

The second proof of concept study was performed under Staudinger conditions using a small inhibitory molecule for the EphB4 receptor. For this purpose, the azide-containing building block **5b** was reacted with **8** [30] to give **9**. The resulting reference compound **9** was obtained after 3 h reaction time in a high yield of 76%. The reaction is shown in Scheme 3.

### Preparation of bioorthogonal ^18^F-containing building blocks

The development of new ^18^F-based radiotracers remains an ongoing goal in the field of radiopharmacy and provides tools for specific cancer diagnostics using positron emission tomography (PET) [31–33]. When radiotracers are based on tumor-specific peptides, proteins or antibodies [34–35], mild and fast methods for radiolabeling are mandatory because of the short half-live of [^18^F]fluoride (*t*_1/2_: 110 min). In most cases, indirect radiolabeling is used for these more sensitive biomacromolecules due to the harsh conditions (i.e., organic solvents, high temperatures and basic conditions) for the direct incorporation of [^18^F]fluoride, which can alter the biological/pharmacological behavior or, at least, destroy sensitive biomolecules [36–38]. For this purpose, bioorthogonal building blocks were developed using Huisgen-click or the Staudinger ligation.

**Scheme 4:**
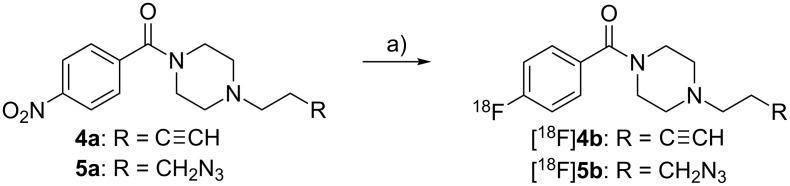
Preparation of the radiolabeling building blocks [^18^F]**4b** and [^18^F]**5b**. Reagents and conditions: a) K[^18^F]F, Kryptofix K 2.2.2, DMSO, 150 °C, 60 min.

**Figure 8:**
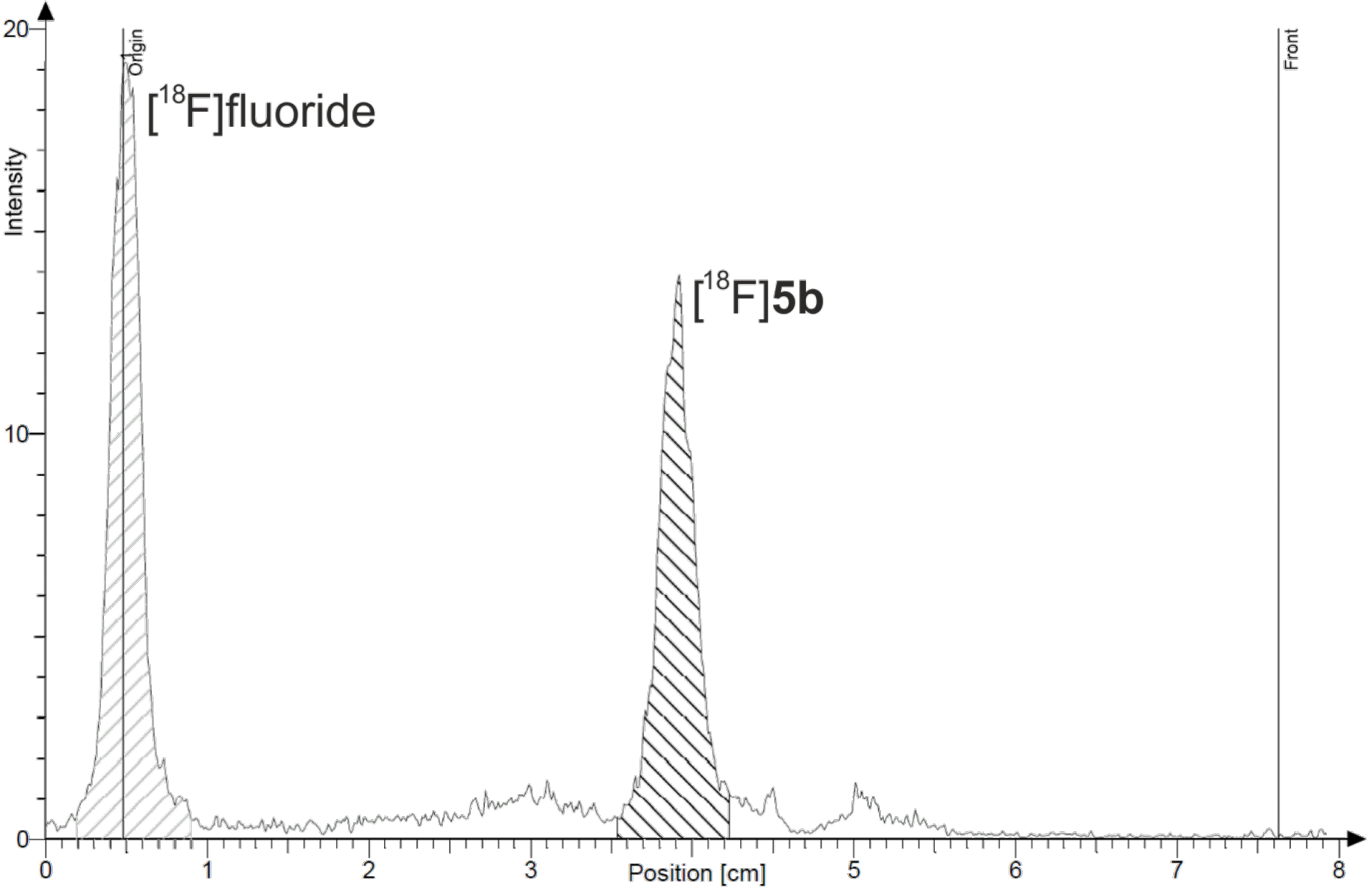
Radio-TLC (eluent: ethanol) of [^18^F]**5b** (*R*_f_ = 0.50; reaction mixture).

**Figure 9:**
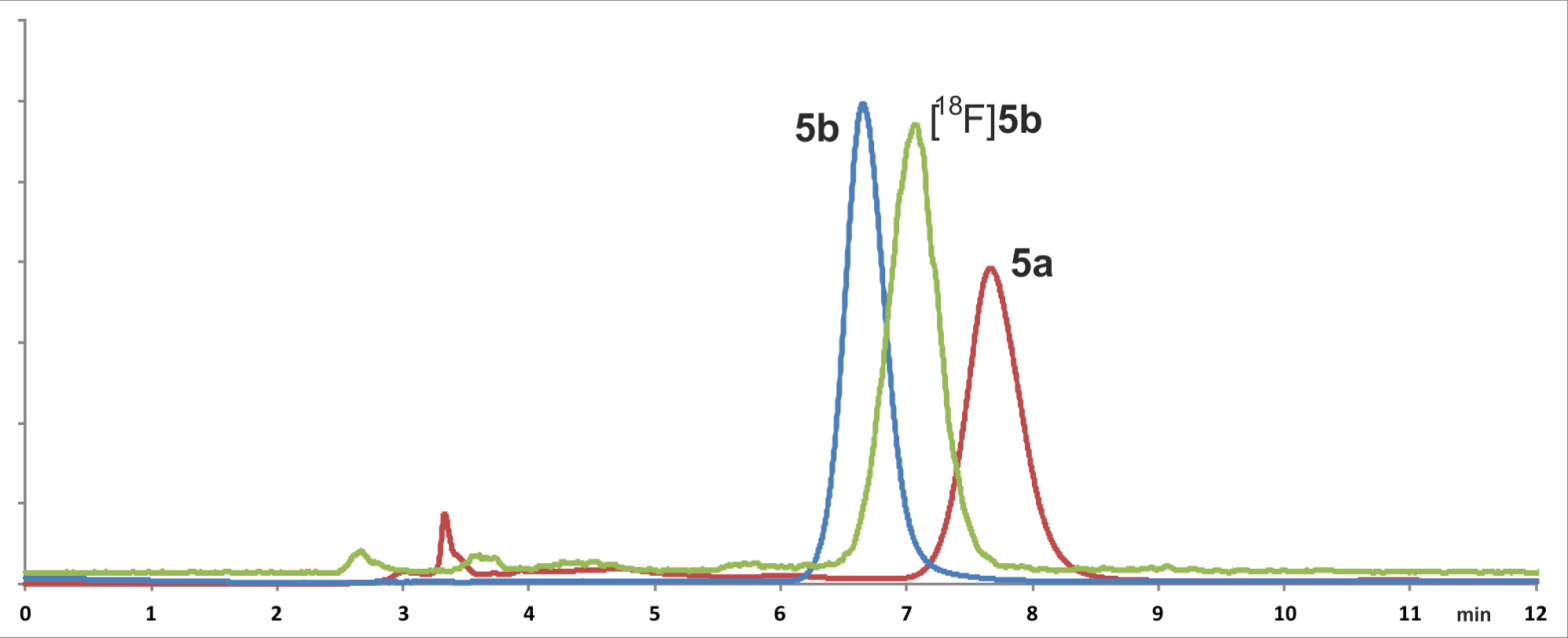
(Radio)HPLC of **5a** (*t*_R_ = 7.7 min, UV trace, red), **5b** (*t*_R_ = 6.7 min, UV trace: blue) and [^18^F]**5b** (*t*_R_ = 7.1 min, γ-trace, green). The differences in the retention times of **5b** and [^18^F]**5b** occurs due to the setup of UV- and γ-detector of the HPLC device.

Using these methods, a synthesis procedure for both bioorthogonal ^18^F-building blocks [^18^F]**4b** and [^18^F]**5b** was developed from their precursors **4a** and **5a**, respectively (Scheme 4). For this purpose, the nitro group of **4b** and **5b** was replaced by [^18^F]fluoride in a nucleophilic aromatic exchange reaction. To evaluate the radiolabeling process, the precursor **4a** (approx. 3–4 mg) was first dissolved in anhydrous acetonitrile and added to dry [^18^F]fluoride (typically 0.5–1 GBq), but the radiochemical yield (RCY) did not exceed 5% after 60 min at 100 °C. Thus, anhydrous DMSO was chosen. After addition of [^18^F]fluoride the resulting mixture was stirred for 30 min at 150 °C and the ^18^F-building blocks were obtained in 12% RCY. An elongation of the reaction time to 60 min afforded [^18^F]**4b** in an elevated RCY of approx. 20% (80–155 MBq, decay corrected) after purification. Similar results were obtained for the labeling of azide-containing [^18^F]**5b** under the same reaction conditions. The radio-TLC of the reaction mixture of [^18^F]**5b** and the appropriate radio-TLC and (radio-)HPLC chromatograms are shown in Figure 8 and Figure 9. Compared to other alkyne and azide functionalized building blocks described in the literature [39], the radiochemical yields and the reaction times of [^18^F]**4b** and [^18^F]**5b** are similar.

Finally, the partition coefficients (log *P*) were determined to be 1.26 ± 0.01 (*n* = 3) for compound **4b** and 1.45 ± 0.01 (*n* = 3) for **5b**. Compared to other standard building blocks like [^18^F]SFB (log *P* = 1.8) [40], FBAM (log *P* = 2.7) [41], 4-fluorobenzaldehyde (log *P* = 1.9) [42] or the click labeling building block p-[^18^F]F-SA (log *P* = 1.7) [43], the lipophilicity of our ^18^F-building blocks is reduced. The hydrophilic character of the building blocks enables a radiolabeling in aqueous solutions and the influence of molecular size of the compounds to be labeled is negligible.

## Conclusion

Novel piperazine derivatives were successfully synthesized with high yields using a convenient synthesis procedure. Evaluation of the NMR spectra showed doubled signals of the NCH_2_ groups of the piperazine moiety. One coalescence point was found in the spectra of the fluorine compounds whereas two different coalescence points were found for nitro compounds. A partial double bond in the amide residue and the limited ring conversion of the amine site led to rotational conformers. Activation energies Δ*G*^#^_exc_ were calculated from coalescence temperatures *T*_c_ which were determined from dynamic ^1^H NMR measurements. The formation of conformers resulting from the partial double bond was additionally determined and verified using single crystal X-ray structure analysis. The second coalescence point is caused by the reduced flipping of the amine part in the nitro compounds. Furthermore, the coalescence temperature for the amine residue is higher than expected for amines. In contrast, the reduced flipping of the amine part was not found for the fluorine compounds. Thus, the coalescence is dependent on the substituent of the benzoyl moiety. Furthermore, a proof of concept was accomplished with a pharmacologically active peptide using the Huisgen-click reaction and compound **4b**. Additionally, **5b** was introduced in a small molecule using the traceless Staudinger ligation. The synthesis of the ^18^F-labeled building blocks [^18^F]**4b** and [^18^F]**5b** was accomplished using the S_N_Ar concept. The appropriate precursors and reference compounds were prepared in two steps from simple commercially available starting materials in high yields, but at this stage the building blocks are not appropriate for further labeling purposes.

## Experimental

### General information

All chemicals were purchased from commercial suppliers and used without further purification unless otherwise specified. Anhydrous THF was purchased from Acros. NMR spectra of all compounds were recorded on an Agilent DD2-400 MHz NMR spectrometer. Chemical shifts of the ^1^H, ^19^F, and ^13^C spectra were reported in parts per million (ppm) using TMS as internal standard for ^1^H/^13^C and CFCl_3_ for ^19^F spectra. Mass spectrometric (MS) data were obtained on a Xevo TQ-S mass spectrometer (Waters) by electron spray ionization (ESI). The melting points were determined on a Galen III melting point apparatus (Cambridge Instruments & Leica) and are uncorrected. Microanalyses were carried out with an LECO CHNS 932 elemental analyzer. Chromatographic separations and TLC detections were performed using Merck Silica Gel 60 (63–200 μm) and Merck Silica Gel 60 F_254_ sheets, respectively. TLCs were developed by visualization under UV light (λ = 254 nm). Diffraction data were collected with a Bruker-Nonius Apex-II-diffractometer using graphite-monochromated Mo K_α_ radiation (λ = 0.71073 Å). The diffraction measurements were performed at −150 °C. The unit cell dimensions were recorded and refined using the angular settings of 7728 reflections for **4b** and 6451 reflections for **3a**. The structures were solved by direct methods and refined against *F*^2^ by full-matrix least-squares using the program suites from G. M. Sheldrick [44–45]. All non-hydrogen atoms were refined anisotropically; all hydrogen atoms except the one attached to the alkyne group (**4b**) were placed on geometrically calculated positions and refined using a riding model. The alkyne-H atom was refined isotropically. CCDC 1479835 and CCDC 1445857 contain the supplementary crystallographic data for compounds **3a** and **4b**. These data can be obtained free of charge from The Cambridge Crystallographic Data Centre via http://www.ccdc.cam.ac.uk/data_request/cif. Analytical (radio-)HPLC was performed on a VWR/Hitachi Elite LaChrome HPLC system, equipped with a reverse-phase column (Nucleosil 100-5C18 Nautilus, Machery Nagel), a UV diode array detector (250 nm), and a scintillation detector (Raytest, Gabi Star) at a flow rate of 1 mL/min. The radioactive compounds were identified with analytical radio-HPLC by comparison of the retention time of the reference compounds. Decay-corrected radiochemical yields (RCYs) were quantified by integration of radioactive peaks on a radio-TLC using a radio-TLC scanner (Fuji, BAS2000). [^18^F]Fluoride was produced using the PET cyclotron Cyclone 18/9 (IBA). [^18^O]H_2_O was irradiated with protons (18 MeV, 30 µA) exploiting the ^18^O(*p*,*n*)^18^F nuclear reaction.

CAUTION! Hazard warning for organic azides: risk of explosion by shock, friction, and fire upon heating. Store these compounds in a cool location.

### Synthetical procedures

**1-(4-Nitrobenzoyl)piperazine (3a):** Piperazine (**2**, 1.0 g, 11.6 mmol) and Et_3_N (1.17g, 11.6 mmol) were dissolved in chloroform (30 mL) and the mixture was cooled to 0 °C. At this temperature, a solution of 4-nitrobenzoyl chloride (**1a**, 1.0 g, 5.4 mmol) dissolved in 30 mL of chloroform was added dropwise and the resulting mixture was allowed to stir at 0 °C for 2 h and at rt for 2 h. Afterwards, the precipitate was filtered and the chloroform solution was washed with saturated hydrogen carbonate solution (30 mL), with water (2 × 30 mL) and dried over Na_2_SO_4_. Finally, the solvent was removed to yield compound **3a** (700 mg, 55%) as yellowish solid. mp 74 °C; ^1^H NMR (400 MHz, CDCl_3_) δ 1.72 (s, 1H, NH), 2.81 (br s, 2H, Pip-H), 2.96 (br s, 2H, Pip-H), 3.33 (br s, 2H, Pip-H), 3.77 (br s, 2H, Pip-H), 7.56 (d, ^3^*J* = 8.7 Hz, 2H, H-o), 8.27 (d, ^3^*J* = 8.7 Hz, 2H, H-m); ^13^C NMR (101 MHz, CDCl_3_) δ 43.5, 46.0, 46.6, 49.0 (4 × Pip-C), 124.0 (C-m), 128.2 (C-o), 142.2 (C-i), 148.5 (C-p), 163.1 (C=O); MS (ESI^+^) *m*/*z* (%): 258 (13) [M^+^ + Na], 236 (100) [M^+^ + H]; Anal. calcd for C_11_H_13_N_3_O_3_ (235.24): C, 56.16; H, 5.57; N, 17.86 %; found, C, 56.41; H, 5.35; N, 17.65.

**1-(4-Fluorobenzoyl)piperazine (3b):** Piperazine (**2**, 2.9 g, 33.67 mmol) was dissolved in HCl (1 M, 50 mL). A solution of 4-fluorobenzoyl chloride (**1b**, 1.1 g, 6.94 mmol) dissolved in acetonitrile (5 mL) was added dropwise and the resulting mixture was stirred for 4 h at rt. Afterwards, additional 9 mL of 1 M HCl were added and the aqueous layer was extracted with ethyl acetate (2 × 20 mL). Then, KOH was added to the aqueous phase until pH 8 was reached. The aqueous layer was again extracted with chloroform (2 × 25 mL), the combined organic layers were dried over Na_2_SO_4_ and the solvent was removed to yield 931 mg of compound **3b**. Spectra are in agreement with those found in the literature [46].

**4-(But-3-yn-1-yl)-1-(4-nitrobenzoyl)piperazine (4a):** Compound **3a** (110 mg, 0.47 mmol), but-3-yn-1-yl tosylate (150 mg, 0.67 mmol) and Et_3_N (100 mg, 0.98 mmol) were dissolved in anhydrous THF (10 mL) and the resulting mixture was stirred at 60 °C for 3 d. After reaction control by TLC, THF was changed by ethyl acetate (15 mL), water (15 mL) was added and the aqueous layer was extracted with ethyl acetate (3 × 15 mL). The combined organic layers were dried over Na_2_SO_4_, the solvent was removed and the crude product was purified via automated column chromatography (Biotage: 10 g KP-Sil, Gradient: petroleum ether → EtOAc) to yield **4a** (112 mg, 84%) as yellow solid. *R*_f_ = 0.59 (ethanol); mp 76 °C; analytical HPLC: *t*_R_ = 6.0 min (eluent: CH_3_CN/H_2_O, 15:85 + 0.1% TFA); ^1^H NMR (400 MHz, CDCl_3_) δ 1.97 (t, ^4^*J* = 2.5 Hz, 1H, ≡CH), 2.36 (dt, ^3^*J* = 7.3 Hz, ^4^*J* = 2.5 Hz, 2H, CH_2_C≡), 2.43 (br s, 2H, Pip-H), 2.51–2.65 (m, 4H, Pip-H, NCH_2_), 3.36 (br s, 2H, Pip-H), 3.79 (br s, 2H, Pip-H), 7.55 (d, ^3^*J* = Hz, 2H, H-o), 8.25 (d, ^3^*J* = Hz, 2H, H-m); ^13^C NMR (101 MHz, CDCl_3_) δ 17.0 (CH_2_C≡), 42.2, 47.6, 52.4, 53.1 (4 × Pip-C), 56.7 (NCH_2_), 69.5 (≡CH), 82.4 (CH_2_C≡), 124.0 (C-m), 128.2 (C-o), 142.0 (C-i), 148.4 (C-p), 167.9 (C=O); MS (ESI^+^) *m*/*z* (%): 288 (100) [M^+^ + H]; Anal. calcd for C_15_H_17_N_3_O_3_ (287.31): C, 62.71; H, 5.96; N, 14.63; found, C, 62.51; H, 5.95; N, 14.65.

**4-(But-3-yn-1-yl)-1-(4-fluorobenzoyl)piperazine (4b):** Compound **3b** (70 mg, 0.34 mmol), but-3-yn-1-yl tosylate (90 mg, 0.40 mmol) and Et_3_N (51 mg, 0.50 mmol) were dissolved in anhydrous THF (6 mL) and the resulting mixture was stirred at 60 °C for 3 d. After reaction control by TLC, THF was changed by ethyl acetate (15 mL), water (15 mL) was added and the aqueous layer was extracted with ethyl acetate (3 × 15 mL). The combined organic layers were dried over Na_2_SO_4_, the solvent was removed and the crude product was purified via automated column chromatography (Biotage: 10 g KP-Sil, Gradient: petroleum ether → EtOAc) to yield **4b** (72 mg, 82%) as colorless solid. *R*_f_ = 0.60 (ethanol); analytical HPLC: *t*_R_ = 5.5 min (eluent: CH_3_CN/H_2_O, 15:85 + 0.1% TFA); ^1^H NMR (400 MHz, CDCl_3_) δ 1.98 (t, ^4^*J* = 2.7 Hz, 1H, ≡CH), 2.38 (dt, ^3^*J* = 7.4 Hz, ^4^*J* = 2.7 Hz, 2H, CH_2_C≡), 2.50 (br s, 4H, Pip-H), 2.62 (t, 2H, ^3^*J* = 7.4 Hz, NCH_2_), 3.45 (br s, 2H, Pip-H), 3.75 (br s, 2H, Pip-H), 7.08 (“t”, ^3^*J*_H,F_ = ^3^*J*_o,m_ = 8.7 Hz, H-m), 7.40 (dd, ^2^*J*_H,F_ = 5.4 Hz, ^3^*J*_o,m_ = 8.7 Hz, H-o); ^13^C NMR (101 MHz, CDCl_3_) δ 17.0 (*C*H_2_C≡), 42.4, 47.8, 52.8 (4 x Pip-C), 56.9 (NCH_2_), 69.4 (≡CH), 82.5 (CH_2_*C*≡), 115.7 (d, ^2^*J*_C,F_ = 21.8 Hz, C-m), 129.5 (d, ^3^*J*_C,F_ = 8.5 Hz, C-o), 131.9 (d, ^4^*J*_C,F_ = 3.3 Hz, C-i), 163.5 (d, ^1^*J*_C,F_ = 249.9 Hz, C-p), 169.5 (C=O); ^19^F NMR (376 MHz, CDCl_3_) δ −110.3; MS (ESI^+^) *m/z* (%): 261 (100) [M^+^ + H]; Anal. calcd for C_15_H_17_FN_2_O (260.31): C, 69.21; H, 6.58; N, 10.76; found, C, 68.99; H, 6.61; N, 10.80.

**4-(3-Azidopropyl)-1-(4-nitrobenzoyl)piperazine (5a):** Compound **3a** (150 mg, 0.64 mmol), 3-azidopropyl tosylate (195 mg, 0.77 mmol) and Et_3_N (129 mg, 1.28 mmol) were dissolved in anhydrous THF (10 mL) and the resulting mixture was stirred at 60 °C for 3 d at rt. After reaction control by TLC, THF was changed by ethyl acetate (15 mL), water (15 mL) was added and the aqueous layer was extracted with ethyl acetate (3 × 15 mL). The combined organic layers were dried over Na_2_SO_4_, the solvent was removed and the crude product was purified via automated column chromatography (Biotage: 10 g KP-Sil, Gradient: petroleum ether → EtOAc) to yield **5a** (176 mg, 87%) as yellow solid. *R*_f_ = 0.49 (ethanol); analytical HPLC: *t*_R_ = 7.7 min (eluent: CH_3_CN/H_2_O, 15:85 + 0.1% TFA); mp 64 °C; ^1^H NMR (400 MHz, CDCl_3_) δ 1.76 (qi, ^3^*J* = 6.8 Hz, 1H, CH_2_), 2.27–2.60 (m, 6H, Pip-H, NCH_2_), 3.25–3.47 (m, 4H, Pip-H, CH_2_N_3_), 3.80 (br s, 2H, Pip-H), 7.56 (d, ^3^*J* = Hz, 2H, H-o), 8.27 (d, ^3^*J* = Hz, 2H, H-m); ^13^C NMR (101 MHz, CDCl_3_) δ 26.2 (CH_2_), 42.3, 47.7, 52.7, 53.5 (4 × Pip-C), 49.5 (CH_2_N_3_), 55.1 (NCH_2_), 124.0 (C-m), 128.2 (C-o), 142.1 (C-i), 148.5 (C-p), 168.0 (C=O); MS (ESI^+^) *m*/*z* (%): 319 (100) [M^+^ + H]; Anal. calcd for C_14_H_18_FN_6_O_3_ (318.33): C, 52.82; H, 5.70; N, 26.40; found, C, 52.79; H, 5.66; N, 26.11.

**4-(3-Azidopropyl)-1-(4-fluorobenzoyl)piperazine (5b):** Compound **3b** (83 mg, 0.40 mmol), 3-azidopropyl tosylate (122 mg, 0.48 mmol) and Et_3_N (61 mg, 0.60 mmol) were dissolved in anhydrous THF (6 mL) and the resulting mixture was stirred at 60 °C for 3 d. After reaction control by TLC, THF was changed by ethyl acetate (15 mL), water (15 mL) was added and the aqueous layer was extracted with ethyl acetate (3 × 15 mL). The combined organic layers were dried over Na_2_SO_4_, the solvent was removed and the crude product was purified via automated column chromatography (Biotage: 10 g KP-Sil, Gradient: petroleum ether → EtOAc) to yield **5b** (94 mg, 81%) as colorless syrup. *R*_f_ = 0,48 (ethanol); analytical HPLC: *t*_R_ = 6.7 min (eluent: CH_3_CN/H_2_O, 15:85 + 0.1% TFA); ^1^H NMR (400 MHz, CDCl_3_) δ 1.76 (qi, 2H, ^3^*J* = 6.7 Hz, CH_2_), 2.30–2.56 (m, 6H, NCH_2_+Pip-H), 3.35 (t, 2H, ^3^*J* = 6.7 Hz, CH_2_N_3_), 3.38–3.88 (br d, 4H, Pip-H), 7.08 (“t”, ^3^*J*_H,F_ = ^3^*J*_o,m_ = 8.8 Hz, H-m), 7.40 (dd, ^2^*J*_H,F_ = 5.4 Hz, ^3^*J_o,m_* = 8.8 Hz, H-o); ^13^C NMR (101 MHz, CDCl_3_) δ 26.3 (CH_2_), 42.4, 48.0 (2 × Pip-C), 49.5 (CH_2_N_3_), 53.0, 53.5 (2 × Pip-C), 55.2 (CH_2_N), 115.7 (d, ^2^*J*_C,F_ = 21.8 Hz, C-m), 129.5 (d, ^3^*J*_C,F_ = 8.4 Hz, C-o), 131.9 (d, ^4^*J*_C,F_ = 3.3 Hz, C-i), 163.5 (d, ^1^*J*_C,F_ = 148.6 Hz, C-p), 163.5 (C=O); ^19^F NMR (376 MHz, CDCl_3_) δ −110.4; MS (ESI^+^) *m*/*z* (%): 292 (100) [M^+^ + H]; Anal. calcd for C_14_H_18_FN_5_O (291.32): C, 57.72; H, 6.23; N, 24.04; found, C, 57.90; H, 6.21; N, 24.24.

**SNEW-peptide (7):** Azide-functionalized peptide **6** (4.55 mg, 2.8 µmol) and **4b** (1.00 mg, 3.84 µmol) were dissolved in PBS buffer (400 µL). Na ascorbate (50 µL, 0.6 M) and CuSO_4_ (50 µL, 0.4 M) were added and the reaction mixture was maintained at 40 °C for 16 h. Peptide **7** was obtained as colorless powder (4.01 mg, 76%) after purification using semi-preparative HPLC and lyophilization; MS (ESI^+^) *m*/*z*: calcd, 1886 [M]^+^; found, 944 [M + 2H]^2+^.

**5-((3-((4-((5-Chlorobenzo[*d*][1,3]dioxol-4-yl)amino)pyrimidin-2-yl)amino)phenyl)sulfonyl)-*N*-(3-(4-(4-fluorobenzoyl)piperazin-1-yl)propyl)pentanamide (9):** Compounds **5b** (63 mg, 0.22 mmol) and **8** (165 mg, 22 mmol) were dissolved in a mixture of acetonitrile and water (5.5 mL, 10:1 v:v) and the resulting solution was maintained at 60 °C for 3 h. Afterwards, the solvent was removed and the crude product was purified via column chromatography to give **9** (112 mg, 69%) as a pale yellow syrup. *R*_f_ = 0.2 (EtOAc); ^1^H NMR (400 MHz, CDCl_3_) δ 1.41–1.52 (m, 2H, CH_2_), 1.57–1.67 (m, 2H, CH_2_), 1.72–1.81 (m, 2H, CH_2_), 1.93–2.00 (t, ^3^*J* = 7.2 Hz, 2H, CH_2_C=O), 2.33–2.54 (m, 6H, 3 × CH_2_N), 2.98 (t, ^3^*J* = 7.6 Hz, 2H, CH_2_S), 3.30–3.89 (m, 6H, 3 × CH_2_N), 5.93 (s, 2H, OCH_2_O), 6.05 (d, ^3^*J* = 5.9 Hz, 1H, H_diox_), 6.69 (d, ^3^*J* = 8.7 Hz, 1H, H_pyr_), 6.92 (d, ^3^*J* = 8.7 Hz, 1H, H_pyr_), 6.97 (s, 1H, NH), 7.07 (t, ^3^*J* = 8.5 Hz, 2H, H-o), 7.38–7.50 (m, 5H, H-m + H_Ar_), 7.97 (d, ^3^*J* = 7.9 Hz, 1H, H_Ar_), 8.05–8.10 (m, 2H, H_diox_ + NH); ^13^C NMR (101 MHz, CDCl_3_) δ 22.0, 22.7, 26.1, 32.8 (4 × CH_2_), 42.3, 47.8 (br. s, 2 × CH_2_Pip), 49.3 (CH_2_N), 52.8, 53.3 (br. s, 2 × CH_2_Pip), 55.0, 55.6 (2 × CH_2_), 97.9 (CH_Ar_), 102.2 (OCH_2_O), 106.7, 115.5 (d, ^2^*J*_C,F_ = 21.5 Hz, C-m), 117.7, 118.6, 120.4, 122.0, 123.3, 123.7, 129.3 (d, ^3^*J*_C,F_ = 8.6 Hz, C-o), 129.5, 131.9 (d, ^4^*J*_C,F_ = 3.3 Hz, C-i), 139.2, 141.2, 143.5, 147.5, 156.9, 159.3, 161.1, 163.3 (d, ^1^*J*_C,F_ = 251.0 Hz, C-p), 169.3 (C=O); ^19^F NMR (376 MHz, CDCl_3_) δ −110.3; MS (ESI^+^) *m*/*z* (%): 752 (80) [M + H]^+^; Anal. calcd for C_36_H_39_ClFN_7_O_6_S (752.25): C 57.48, H 5.23, N 13.03; found, C 57.66, H 5.21, N 13.31.

**4-(But-3-yn-1-yl)-1-(4-[^18^F]fluorobenzoyl)piperazine ([^18^F]4b):** Similar as described in [47] an anion-exchange cartridge (Waters, Sep-Pak Light Accell Plus QMA) was activated by rinsing with 5 mL of a 1 M NaHCO_3_ solution and 10 mL of deionized H_2_O. It was charged with [^18^F]fluoride (0.5–1 GBq) and eluted with 1.5 mL of a solution of Kryptofix 2.2.2 (10 mg/mL) and K_2_CO_3_ (13 mM) in 7 mL CH_3_CN and 43 mL H_2_O. The solvents were evaporated azeotropically by subsequent addition of three portions of 1 mL each of anhydrous CH_3_CN under a stream of nitrogen at 110 °C. Precursor **4a** (3–4 mg) was dissolved in 400 µL of anhydrous DMSO, and the mixture was added to the [^18^F]fluoride-containing sealed vial. The resulting solution was heated at 150 °C for 60 min. Afterwards, the mixture was treated with 25 mL deionized H_2_O and then passed through a C18 cartridge (LiChrolut RP-18/Merck, 500 mg) to yield 80–155 MBq (≈20% RCY, dc) of [^18^F]**4b**. Samples for analytical radio-TLC and radio-HPLC were taken. Analytical radio-TLC: *R*_f_ = 0.50 (ethanol). Analytical radio-HPLC: *t*_R_ = 5.7 min (eluent: CH_3_CN/H_2_O, 15:85 + 0.1% TFA).

**4-(3-Azidopropyl)-1-(4-[^18^F]fluorobenzoyl)piperazine ([^18^F]5b):** Similar as described in [47] an anion-exchange cartridge (Waters, Sep-Pak Light Accell Plus QMA) was activated by rinsing with 5 mL of a 1 M NaHCO_3_ solution and 10 mL of deionized H_2_O. It was charged with [^18^F]fluoride (0.5–1 GBq) and eluted with 1.5 mL of a solution of Kryptofix 2.2.2 (10 mg/mL) and K_2_CO_3_ (13 mM) in 7 mL CH_3_CN and 43 mL H_2_O. The solvents were evaporated azeotropically by subsequent addition of three portions of 1 mL each of anhydrous CH_3_CN under a stream of nitrogen at 110 °C. Precursor **5a** (3–4 mg) was dissolved in 400 µL of anhydrous DMSO, and the mixture was added to the [^18^F]fluoride-containing sealed vial. The resulting solution was heated at 150 °C for 60 min. Afterwards, the mixture was treated with 25 mL deionized H_2_O and then passed through a C18 cartridge (LiChrolut RP-18/Merck, 500 mg) to yield 69–134 MBq (≈17% RCY, dc) of [^18^F]**5b**. Samples for analytical radio-TLC and radio-HPLC were taken. Analytical radio-TLC: *R*_f_ = 0.49 (ethanol). Analytical radio-HPLC: *t*_R_ = 7.1 min (eluent: CH_3_CN/H_2_O, 15:85 + 0.1% TFA).

## Supporting Information

File 1 Copies of NMR spectra of investigated piperazines, radioHPLC chromatograms, and separation methods for piperazines.
