# Correction: Synthesis, dynamic NMR characterization and XRD studies of novel *N*,*N*’-substituted piperazines for bioorthogonal labeling

**DOI:** 10.3762/bjoc.13.32

**Published:** 2017-02-15

**Authors:** Constantin Mamat, Marc Pretze, Matthew Gott, Martin Köckerling

**Affiliations:** 1 Helmholtz-Zentrum Dresden-Rossendorf, Institut für Radiopharmazeutische Krebsforschung, Bautzner Landstraße 400, D-01328 Dresden, Germany; 2 Technische Universität Dresden, Fachrichtung Chemie und Lebensmittelchemie, D-01062 Dresden, Germany; 3 Medizinische Fakultät Mannheim der Universität Heidelberg, Institut für Klinische Radiologie und Nuklearmedizin, Theodor-Kutzner-Ufer 1-3, D-68167 Mannheim, Germany; 4 Universität Rostock, Institut für Chemie – Festkörperchemie, Albert-Einstein-Straße 3a, D-18059 Rostock, Germany

**Keywords:** building blocks, coalescence, dynamic NMR, labeling, Staudinger ligation

In the original article an incorrect caption for Figure 1 was given. The assignment of the solvents to the capital letters was not correct. The correct caption of Figure 1 is: ^1^H NMR spectra of compound **3a** measured in five different solvents: (A) acetonitrile-*d*_3_, (B) methanol-*d*_4_, (C) acetone-*d*_6_, (D) DMSO-*d*_6_ and (E) CDCl_3_ (orange: region of interest). Figure 1 with the corrected caption is given below.

**Figure 1:**
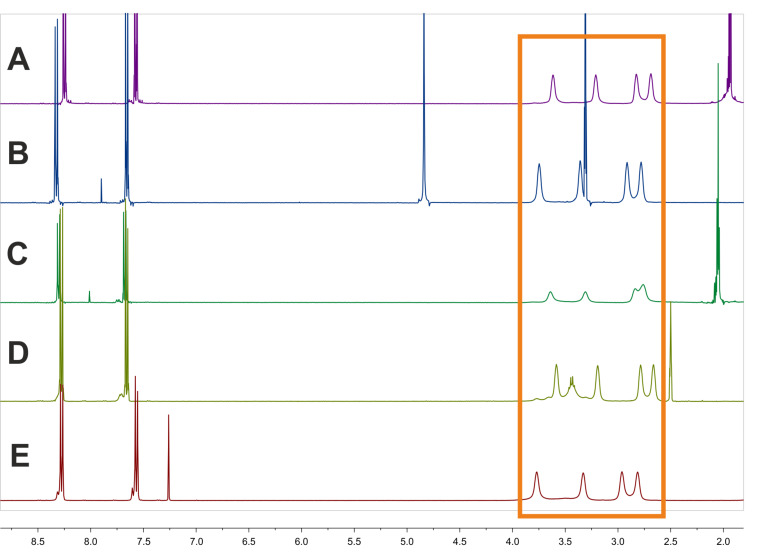
^1^H NMR spectra of compound **3a** measured in five different solvents: (A) acetonitrile-*d*_3_, (B) methanol-*d*_4_, (C) acetone-*d*_6_, (D) DMSO-*d*_6_ and (E) CDCl_3_ (orange: region of interest).

