# Supplementary material for: Synthesis, dynamic NMR characterization and XRD studies of novel N,N’-substituted piperazines for bioorthogonal labeling
Source: Beilstein J Org Chem. 2016 Nov 21;12:2478–89. doi: 10.3762/bjoc.12.242 (PMC5238536; doi:10.3762/bjoc.12.242)

Supporting Information
for

Synthesis, dynamic NMR characterization and XRD studies of novel *N*,*N'*-substituted piperazines for bioorthogonal labeling

Constantin Mamat*[1,2], Marc Pretze[3], Matthew Gott [1] and Martin Köckerling[4]

Address: [1]Helmholtz-Zentrum Dresden-Rossendorf, Institut für Radiopharmazeutische Krebsforschung, Bautzner Landstraße 400, D-01328 Dresden, Germany, [2]Technische Universität Dresden, Fachrichtung Chemie und Lebensmittelchemie, D-01062 Dresden, Germany, [3]Medizinische Fakultät Mannheim der Universität Heidelberg, Institut für Klinische Radiologie und Nuklearmedizin, Theodor-Kutzner-Ufer 1-3, D-68167 Mannheim, Germany and [4]Universität Rostock, Institut für Chemie – Festkörperchemie, Albert-Einstein-Straße 3a, D-18059 Rostock, Germany

Email: Constantin Mamat - c.mamat@hzdr.de

* Corresponding author

Table of Contents

NMR spectra of compounds ………………………………………………………………………….. S2

Dynamic NMR spectra of **3a**, **3b**, **4a** and **5a** …………………………………………………….…… S12

Radiochemistry ……………………………………………………………………………………..…. S14

Chromatographic purifications of compounds **4a**, **4b**, **5a** and **5b** using BIOTAGE ………………… S18

NMR Spectra of compounds

^{1}H NMR of compound **3a**

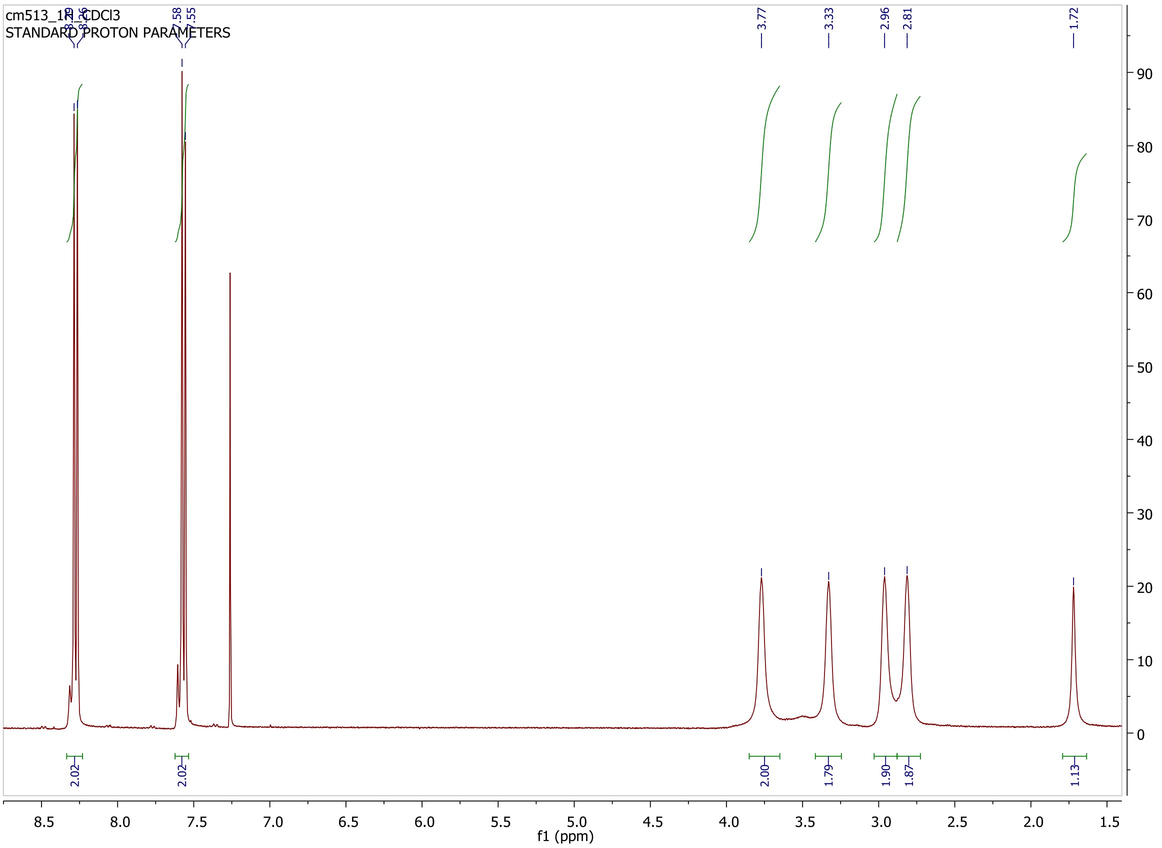

H,H-COSY of compound **3a**

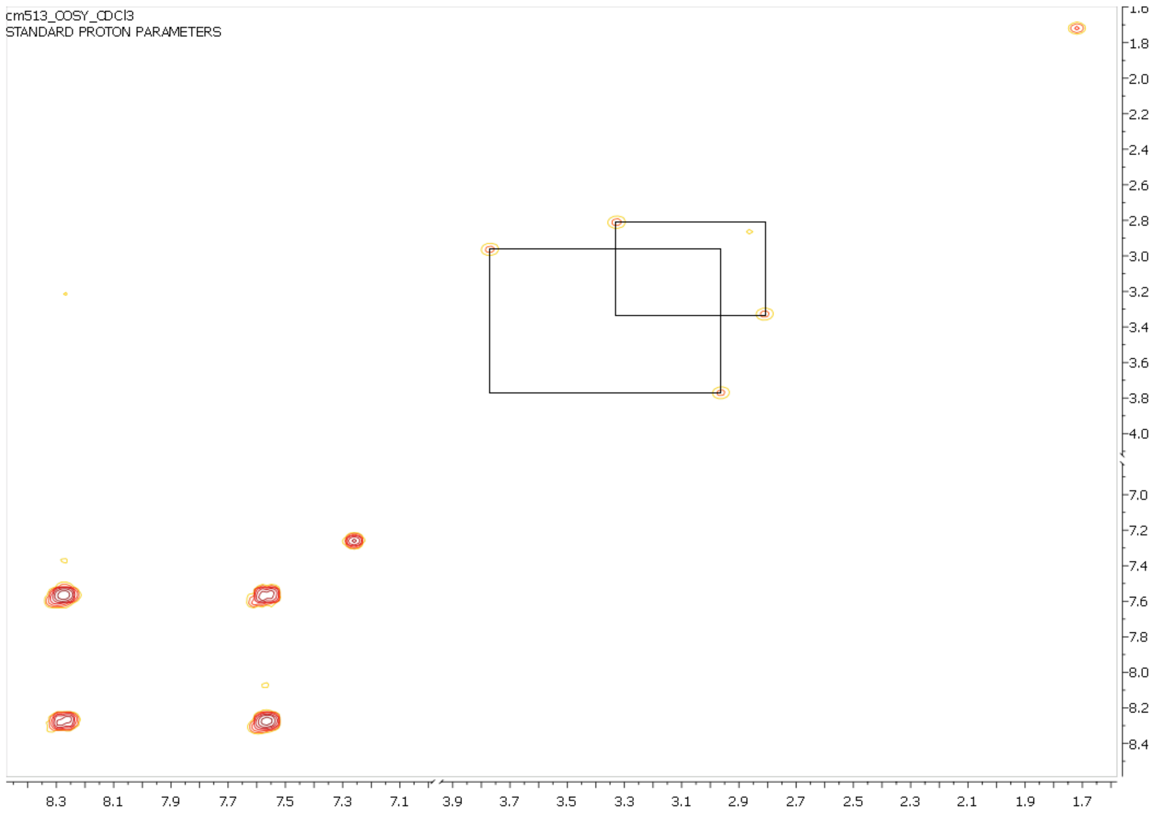

^{13}C NMR of compound **3a**

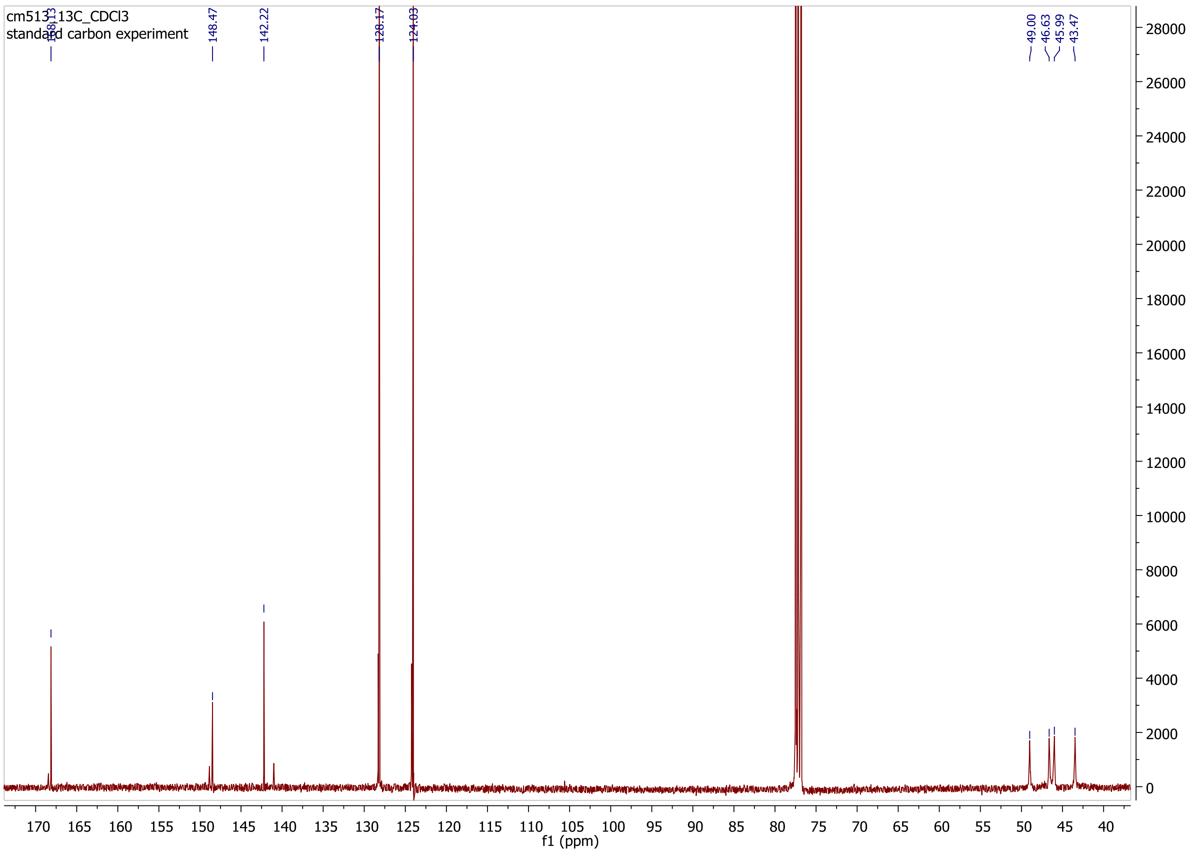

HSQC of compound **3a**

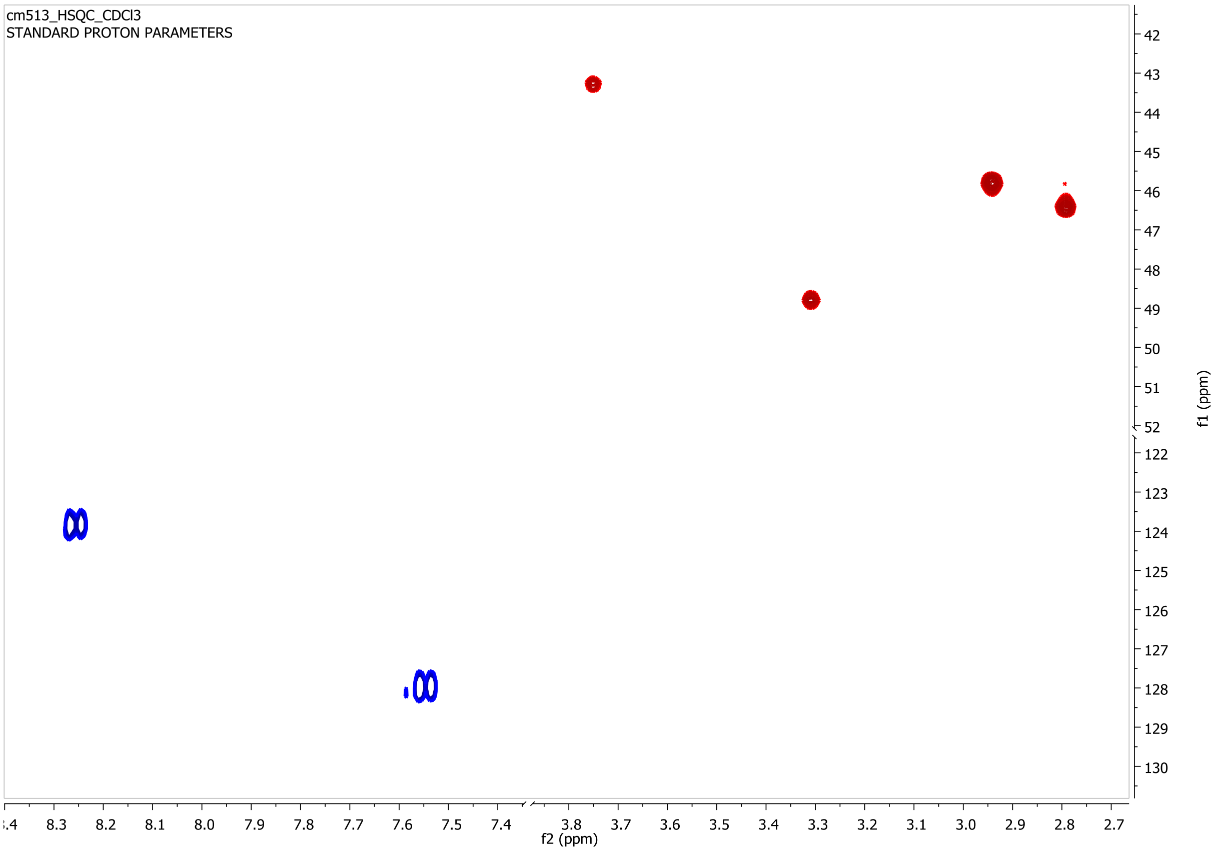

^{1}H NMR of compound **3b**

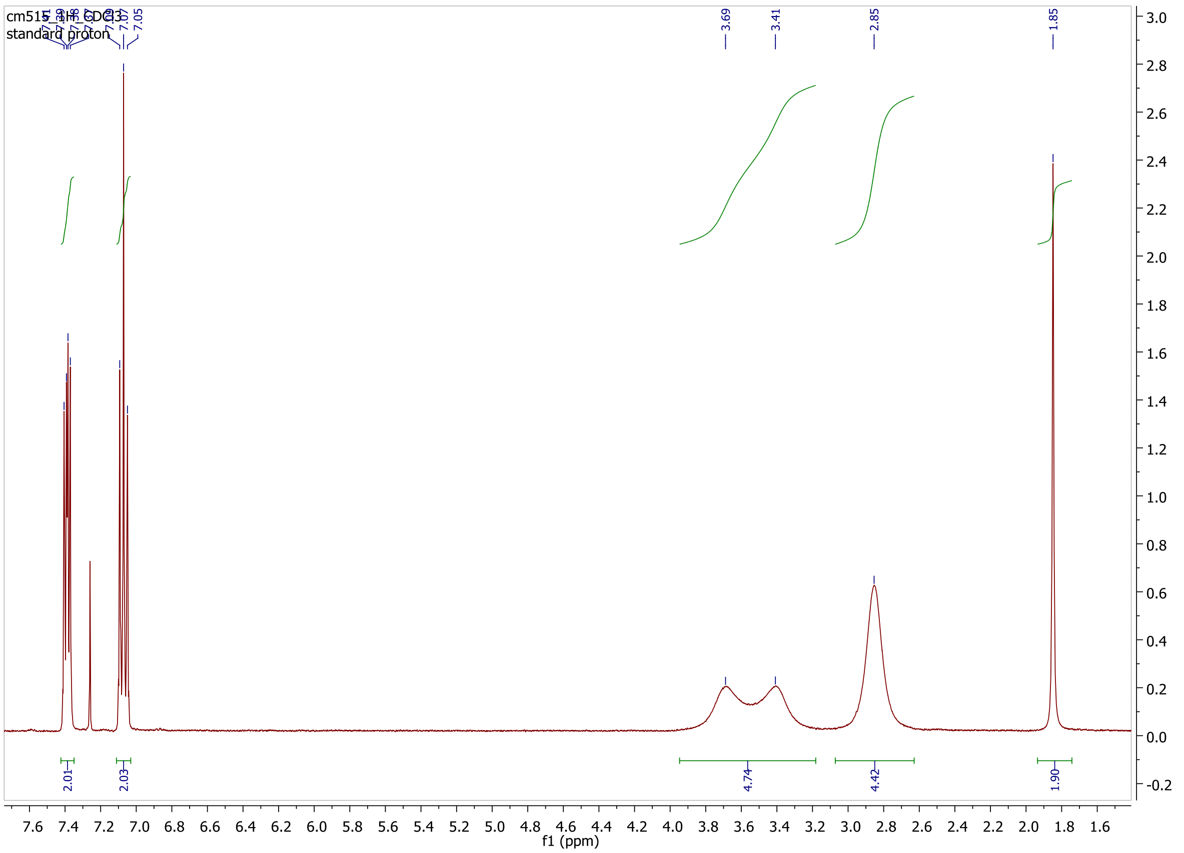

^{13}C NMR of compound **3b**

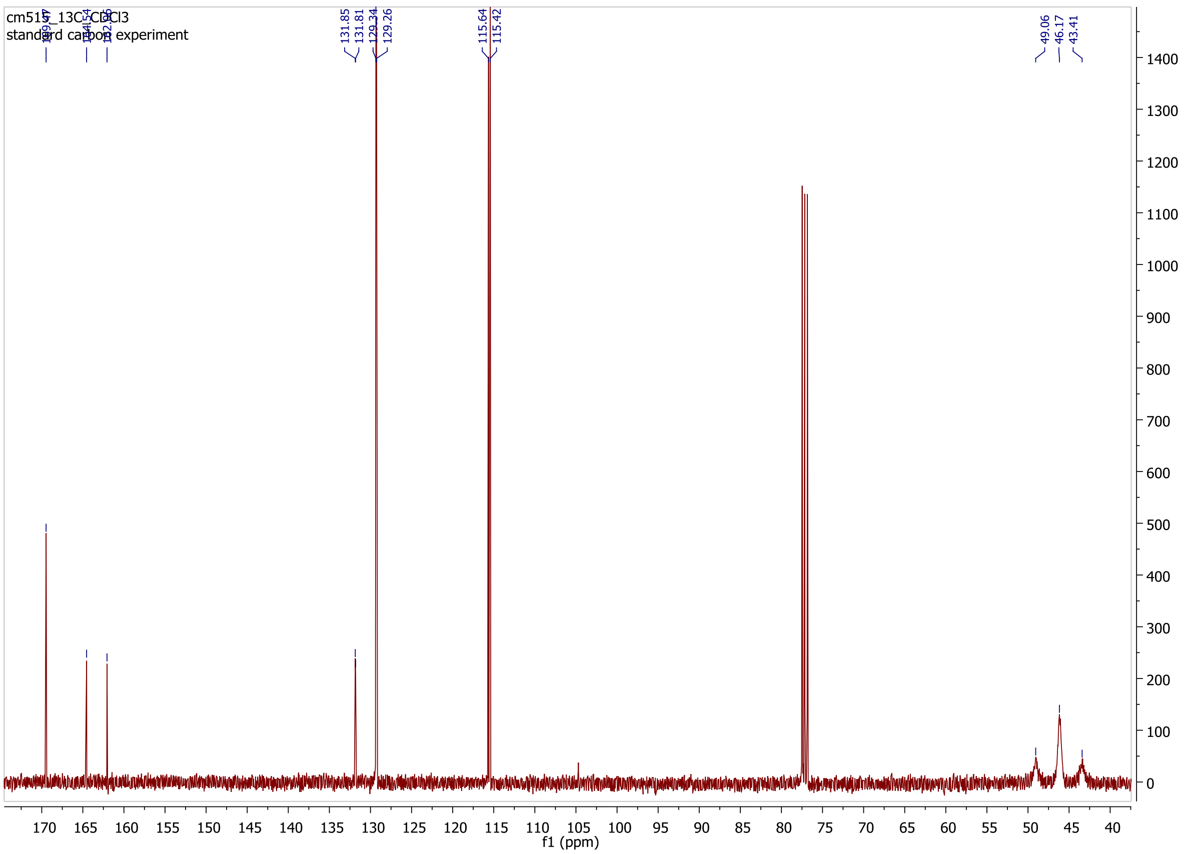

^{1}H NMR of compound **4a**

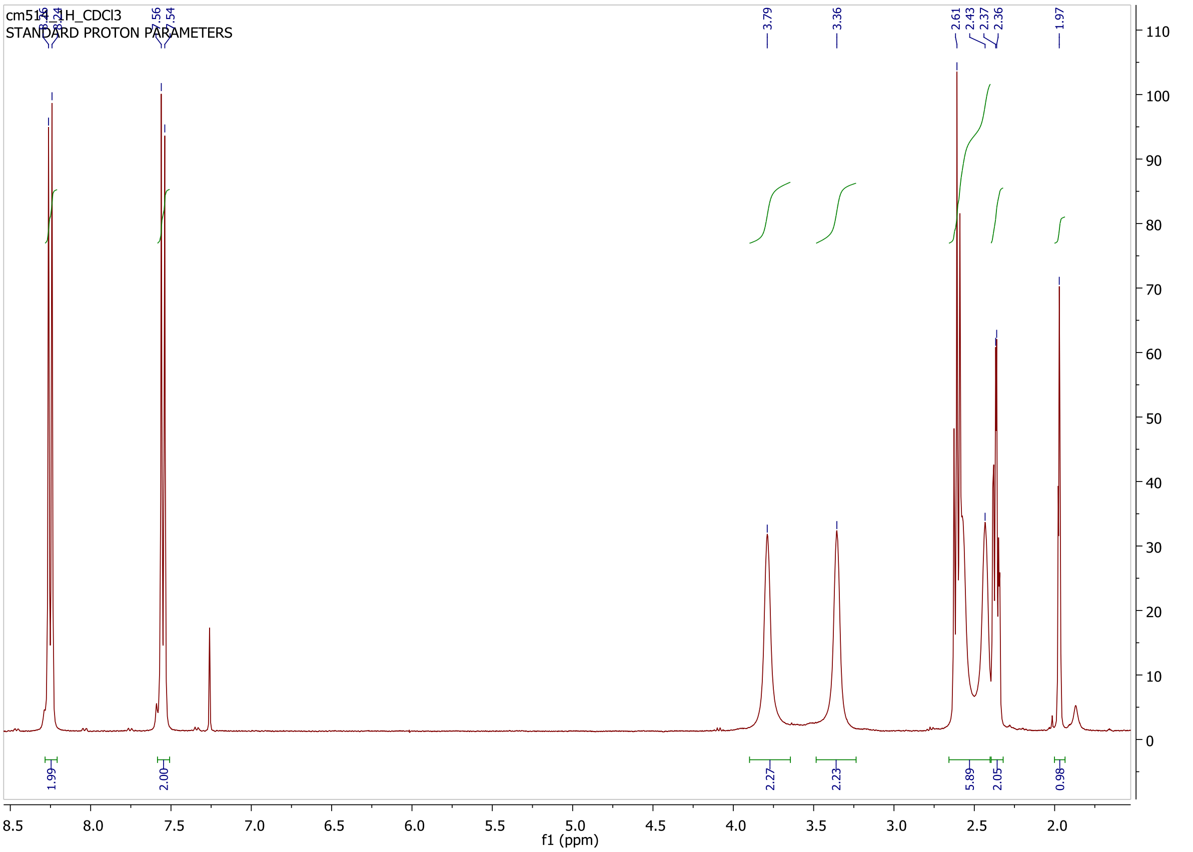

^{13}C NMR of compound **4a**

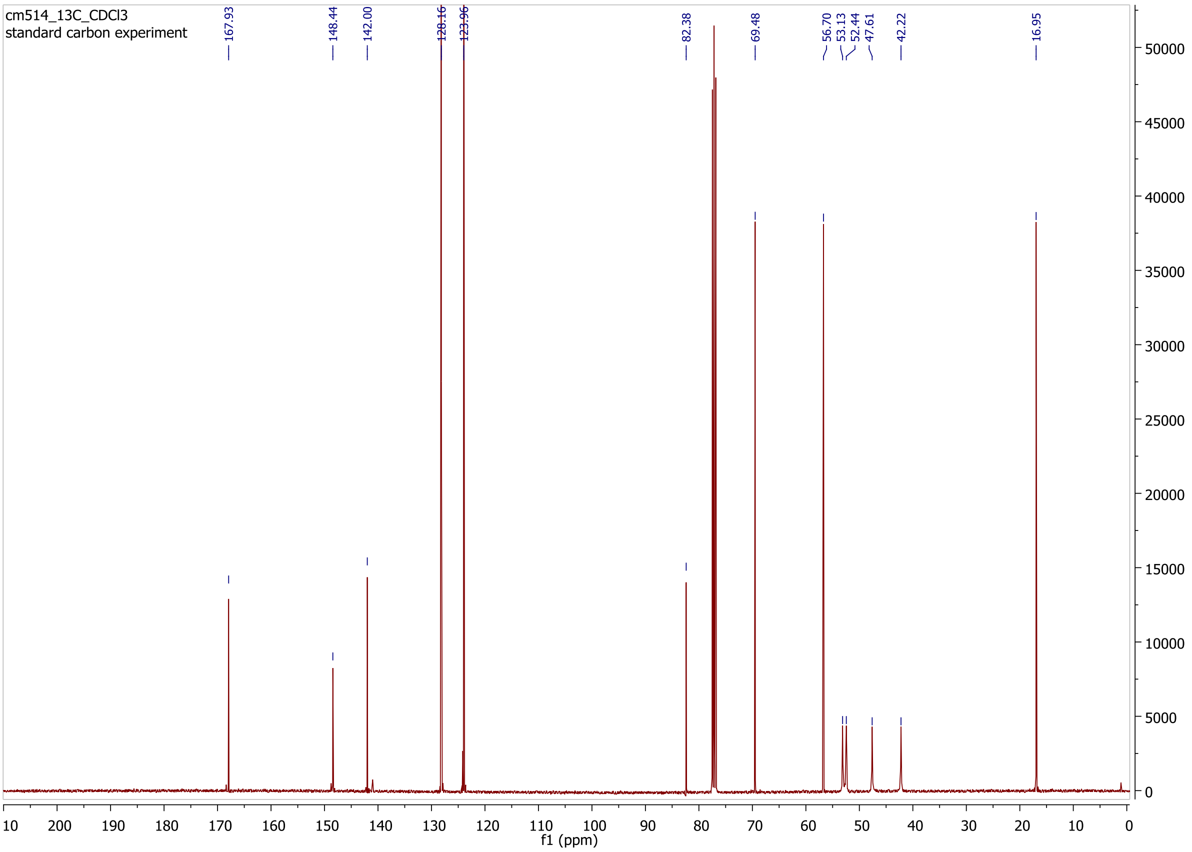

HSQC of compound **4a**

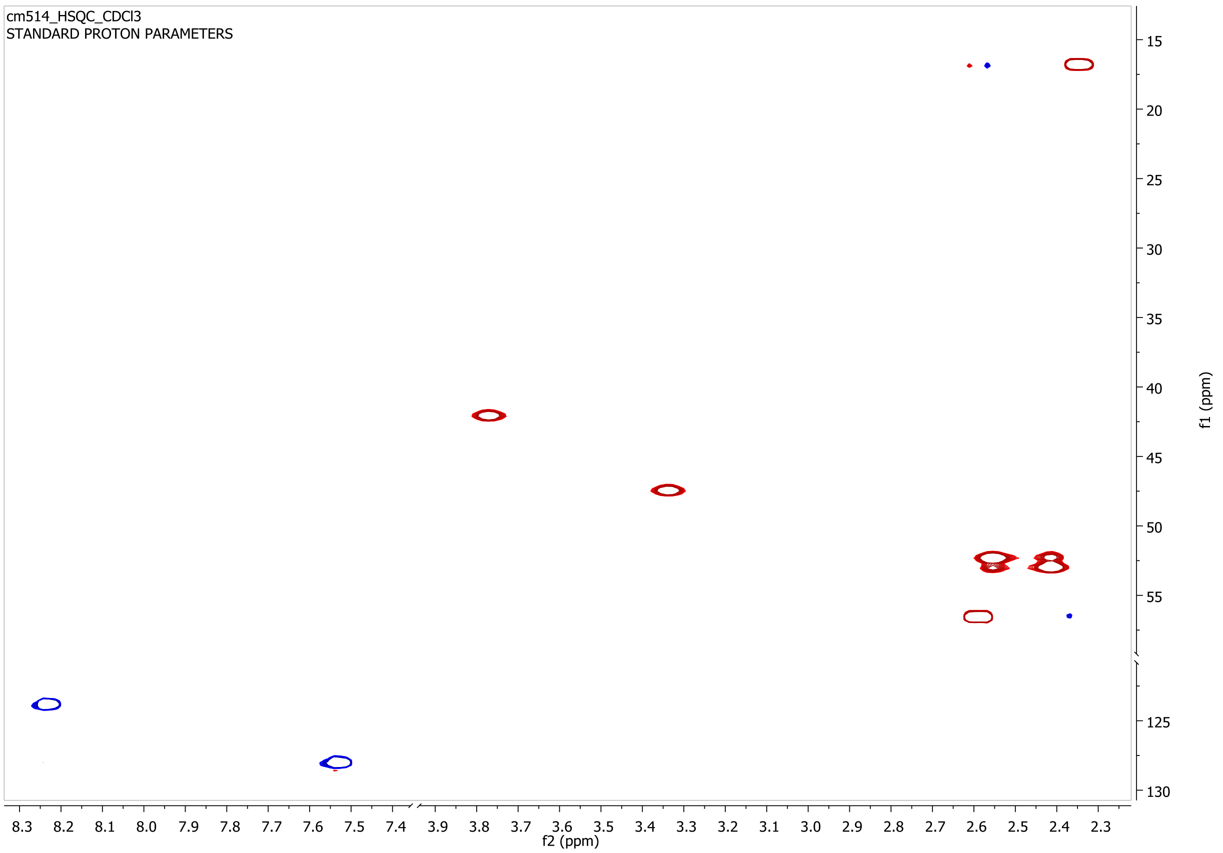

^{1}H NMR of compound **4b**

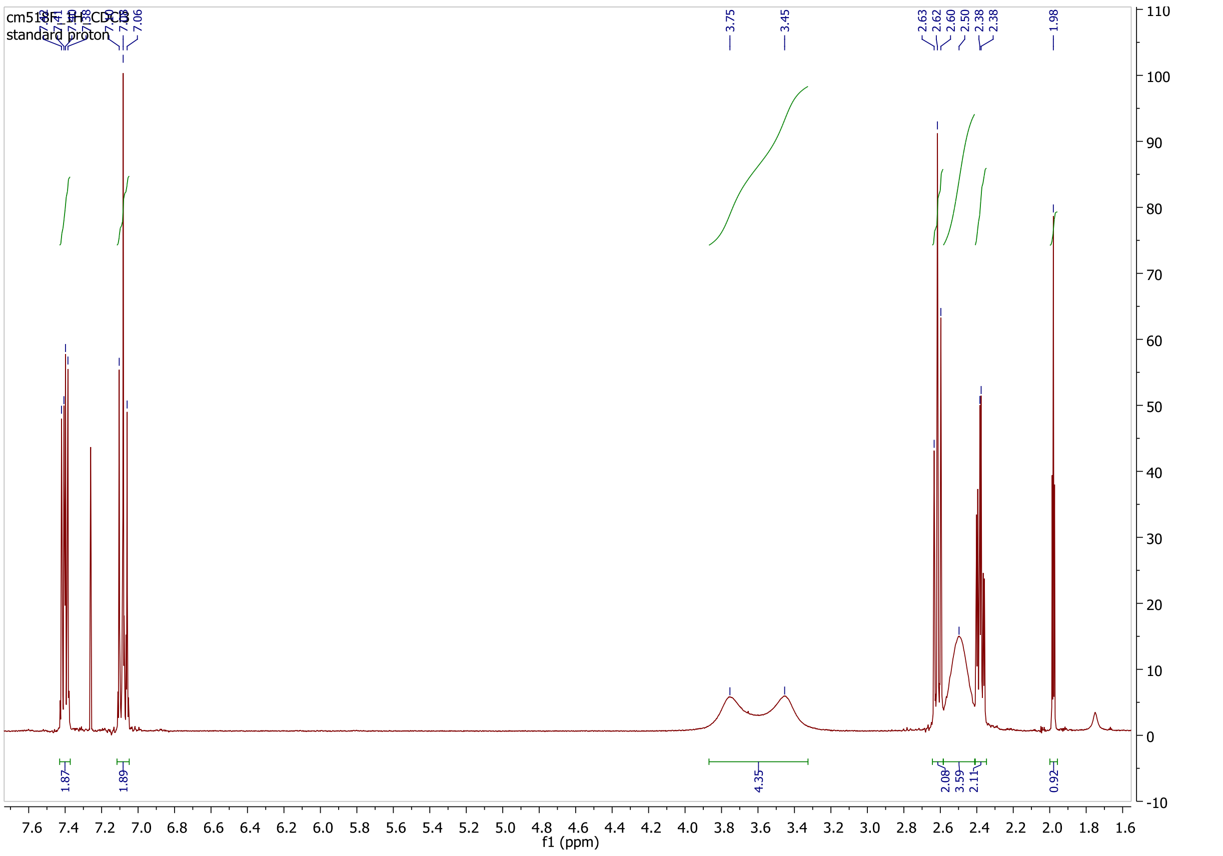

H,H-COSY of compound **4b**

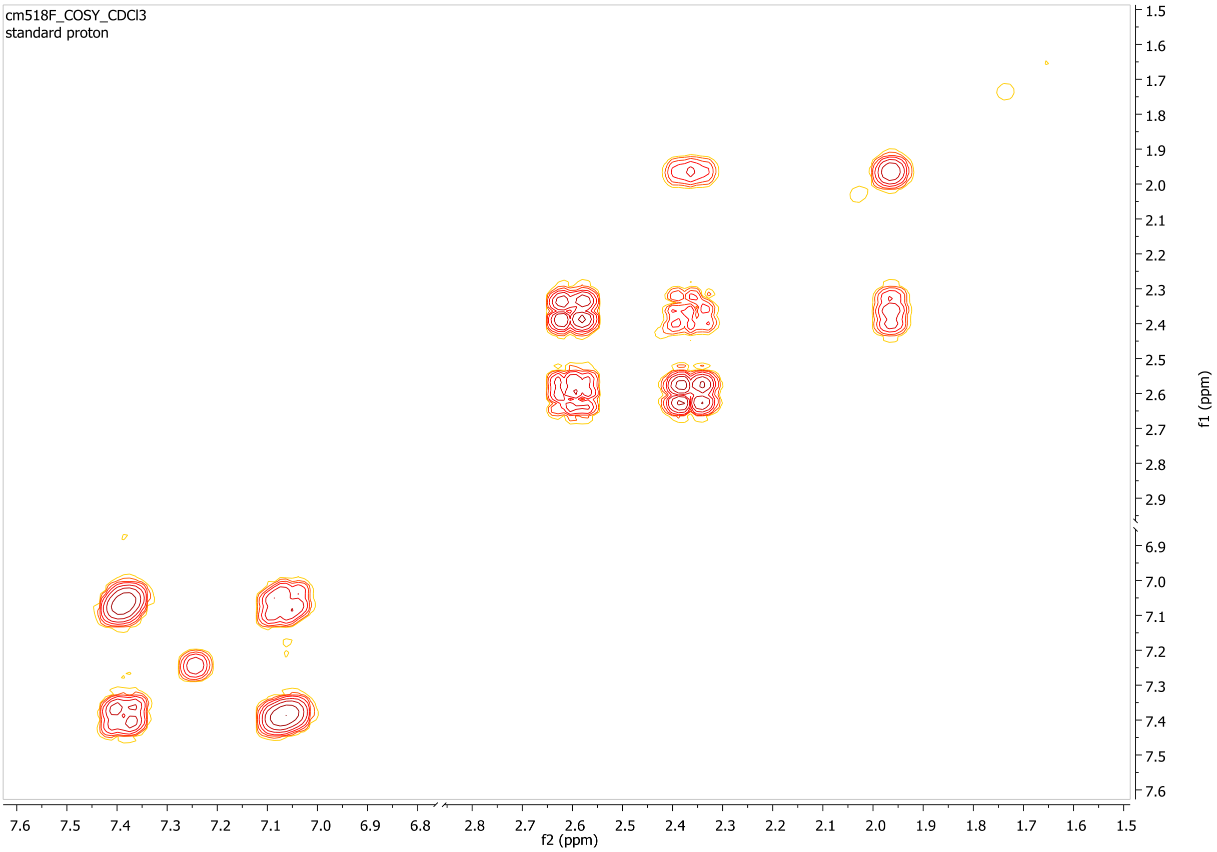

^{13}C NMR of compound **4b**

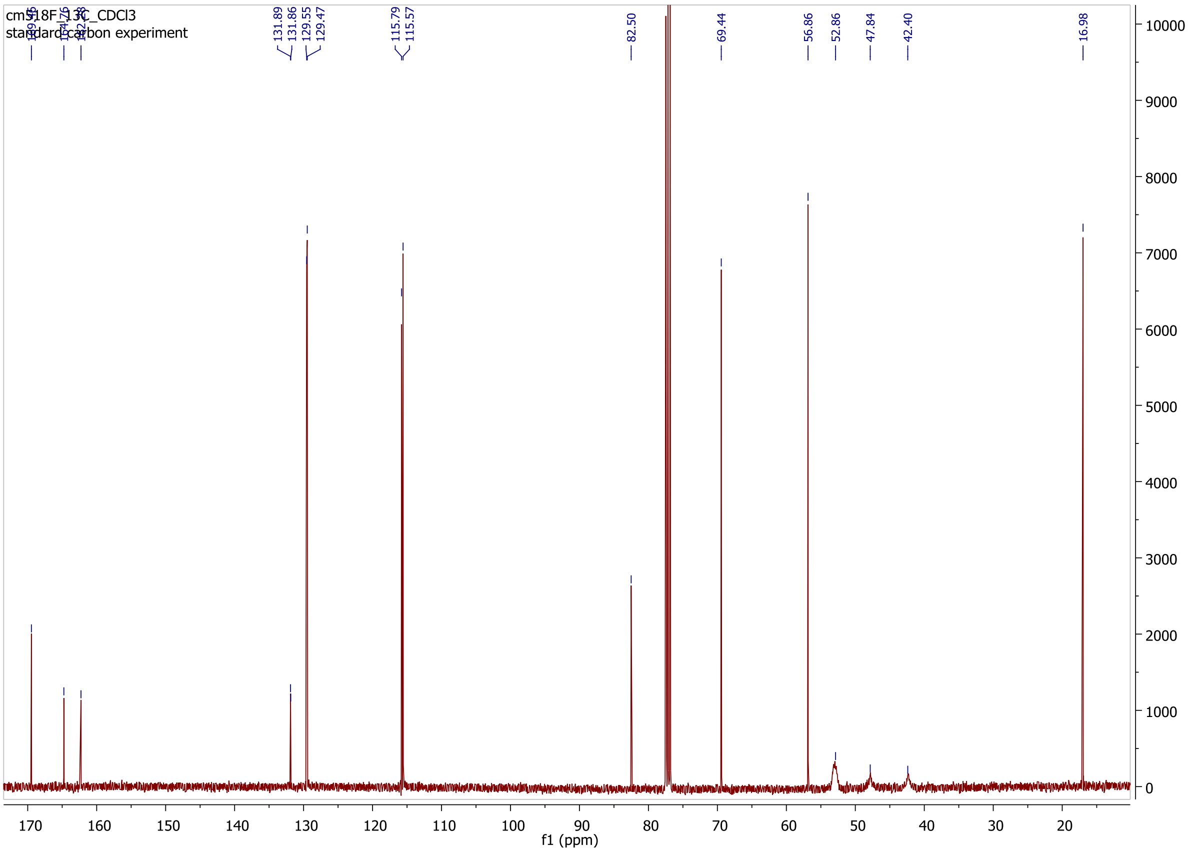

HSQC of compound **4b**

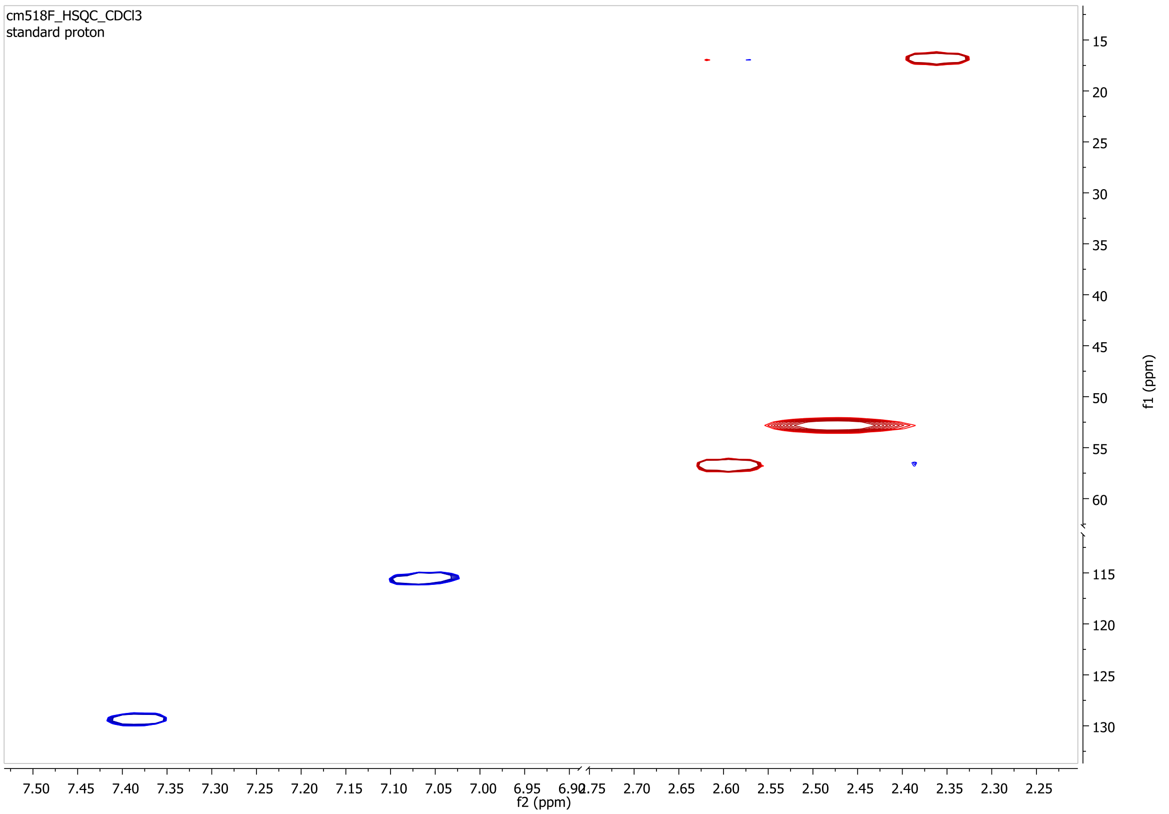

^{1}H NMR of compound **5a**

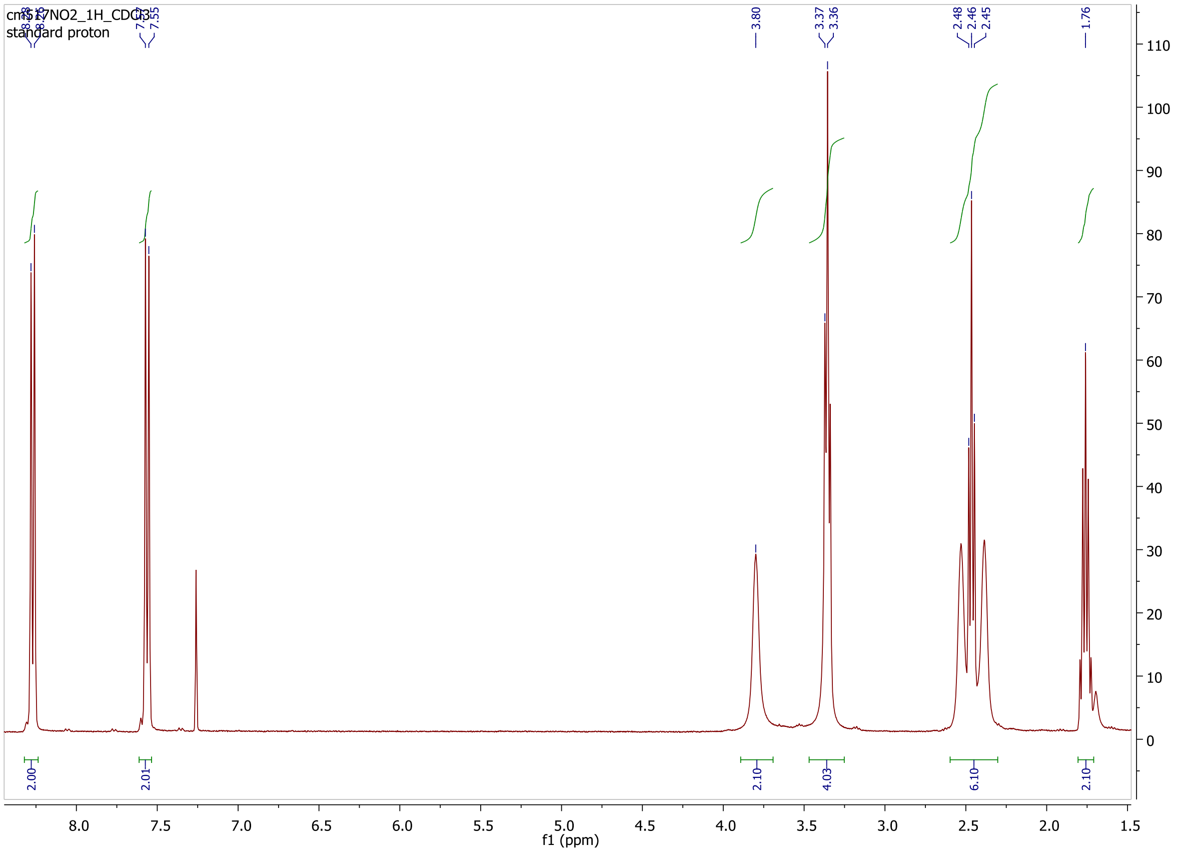

H,H-COSY of compound **5a**

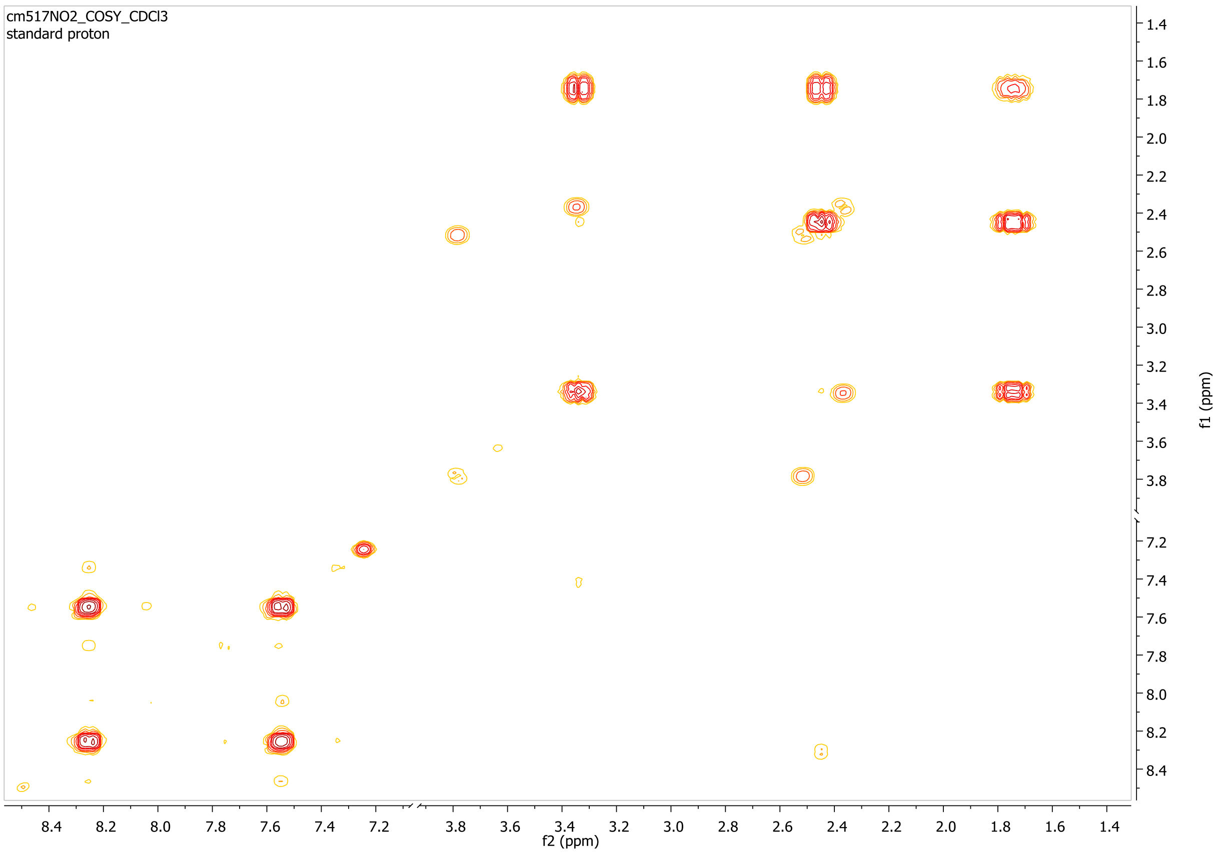

^{13}C NMR of compound **5a**

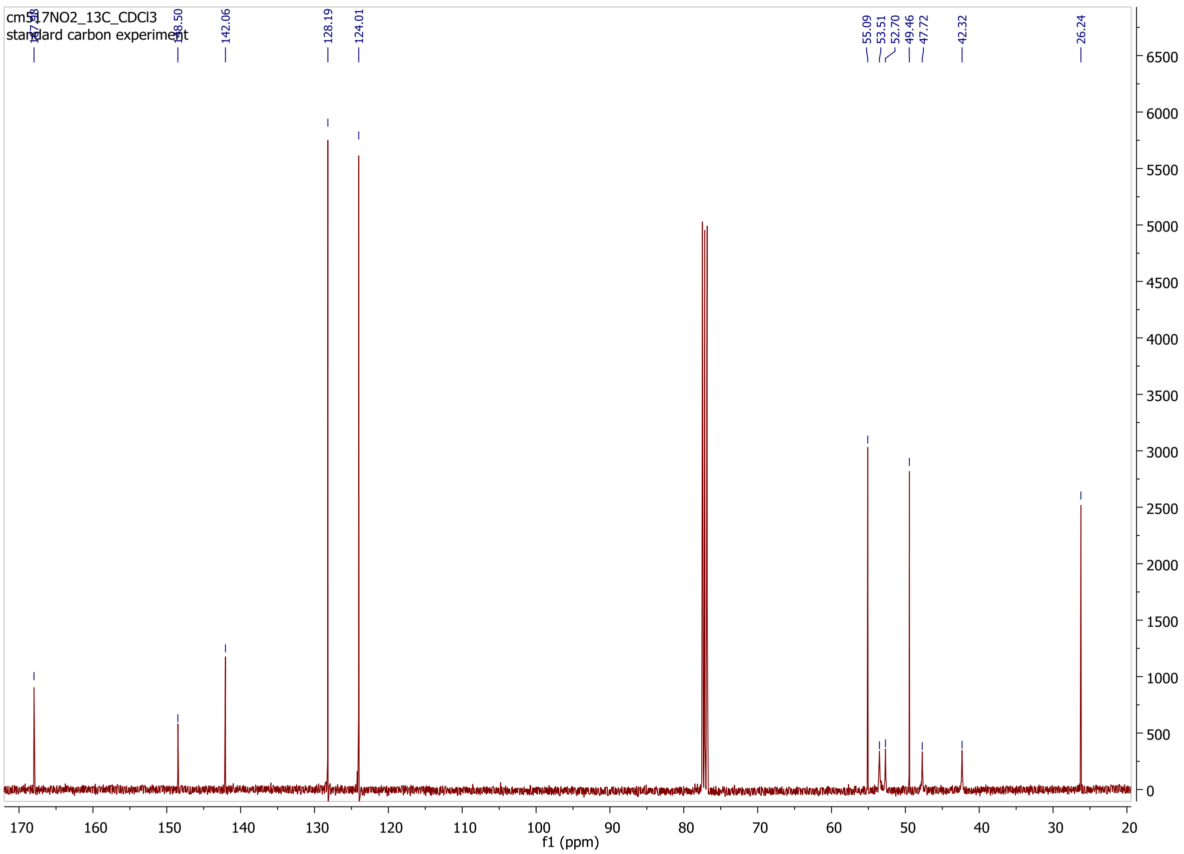

HSQC of compound **5a**

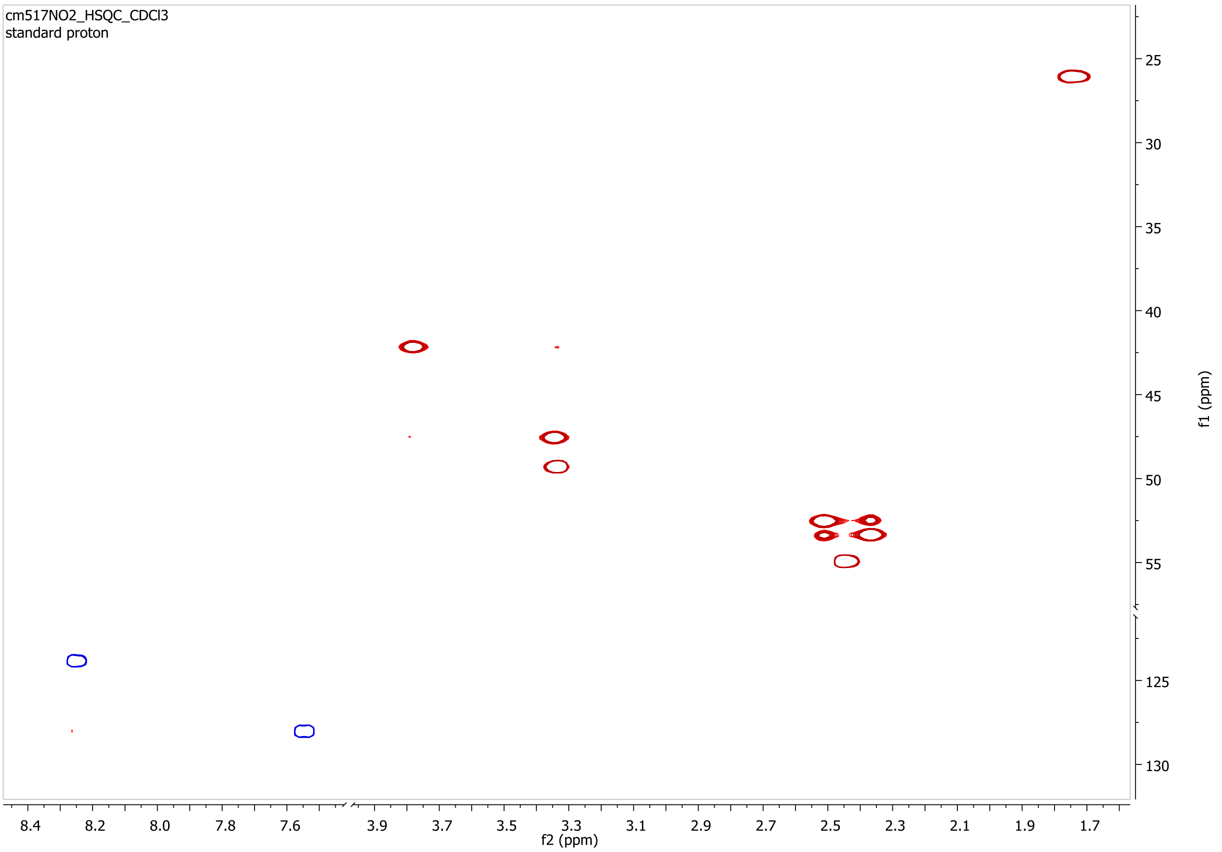

^{1}H NMR of compound **5b**

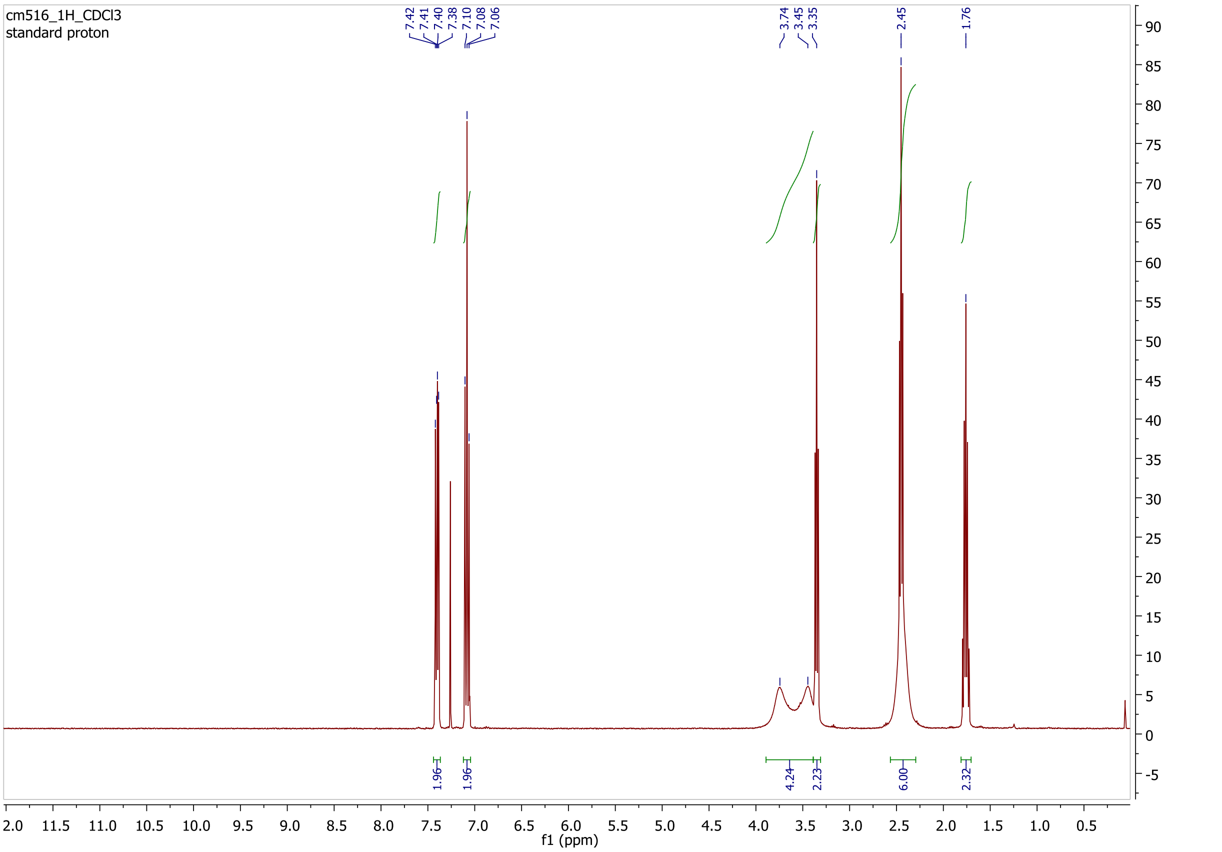

^{13}C NMR of compound **5b**

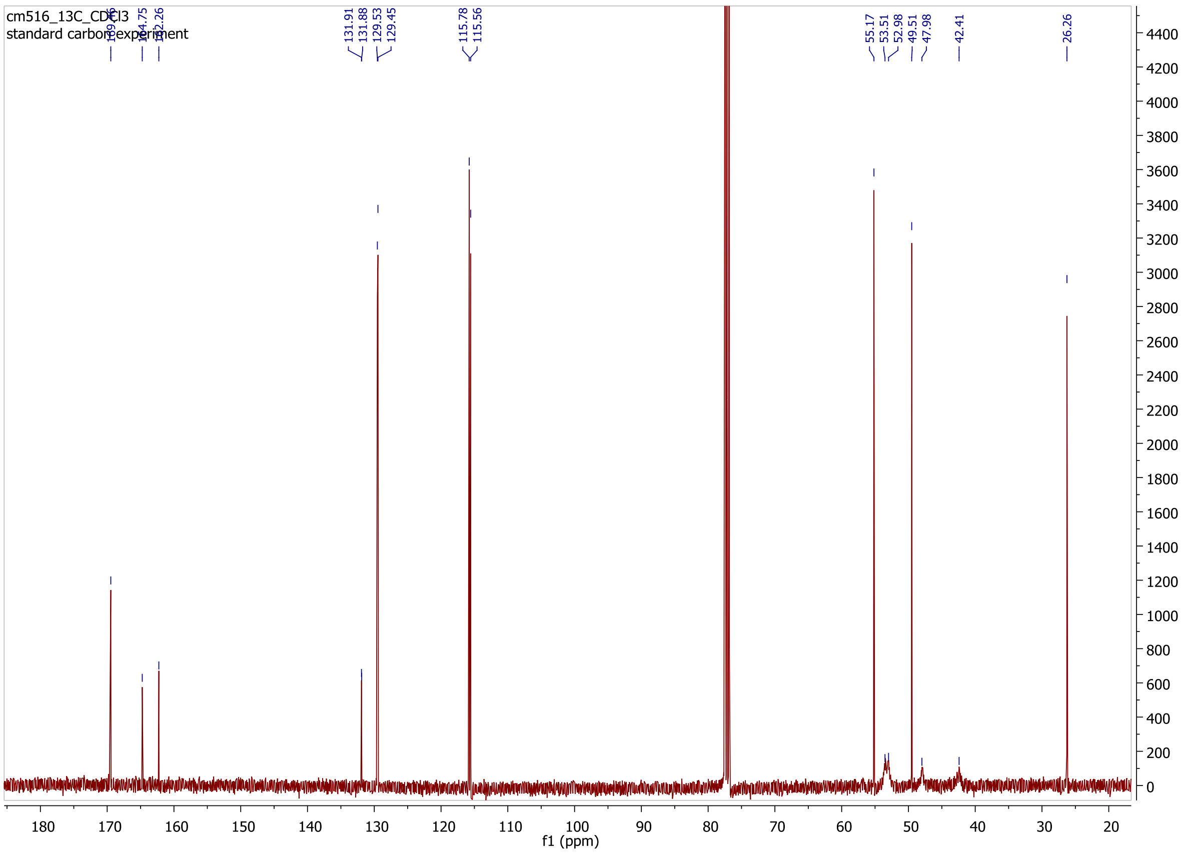

Dynamic NMR spectra

^{1}H NMR of **3a** measured at various temperatures (25–100 °C) in DMSO-d_6

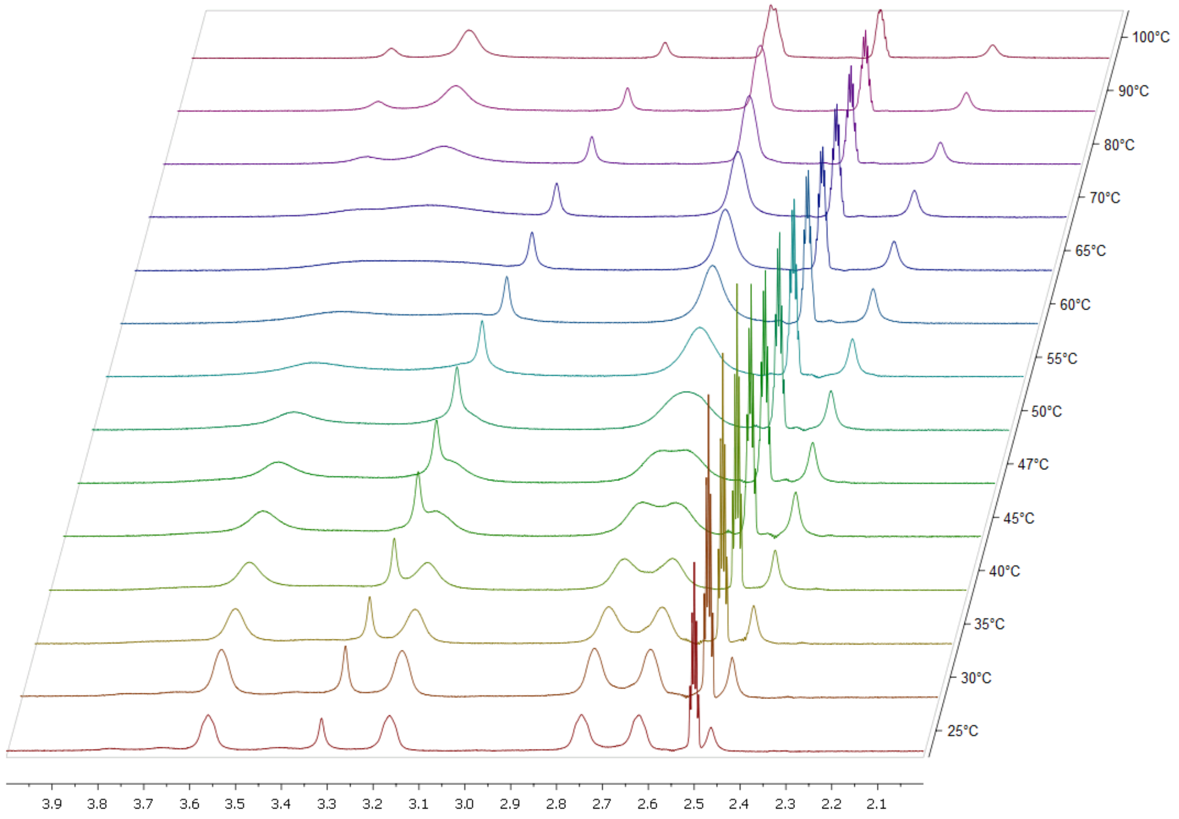

^{1}H NMR of **3b** measured at various temperatures (21–70 °C) in DMSO-d_6

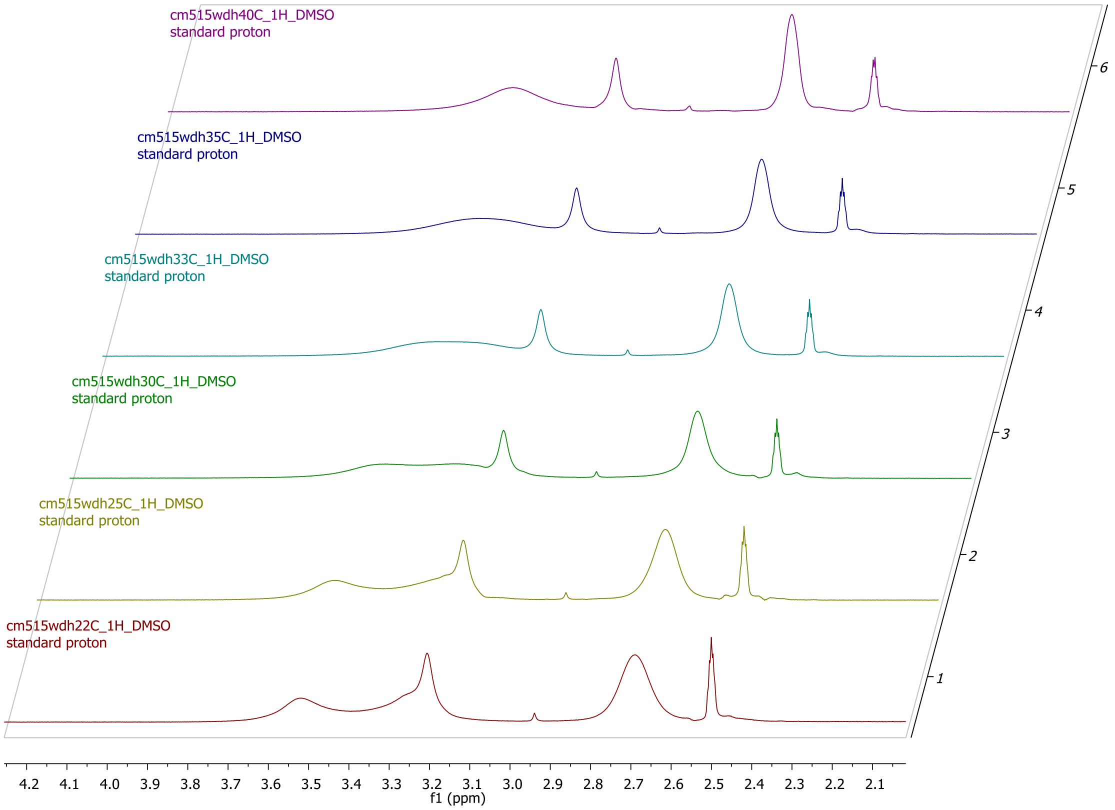

^{1}H NMR of **4a** measured at various temperatures (25–100 °C) in DMSO-d_6

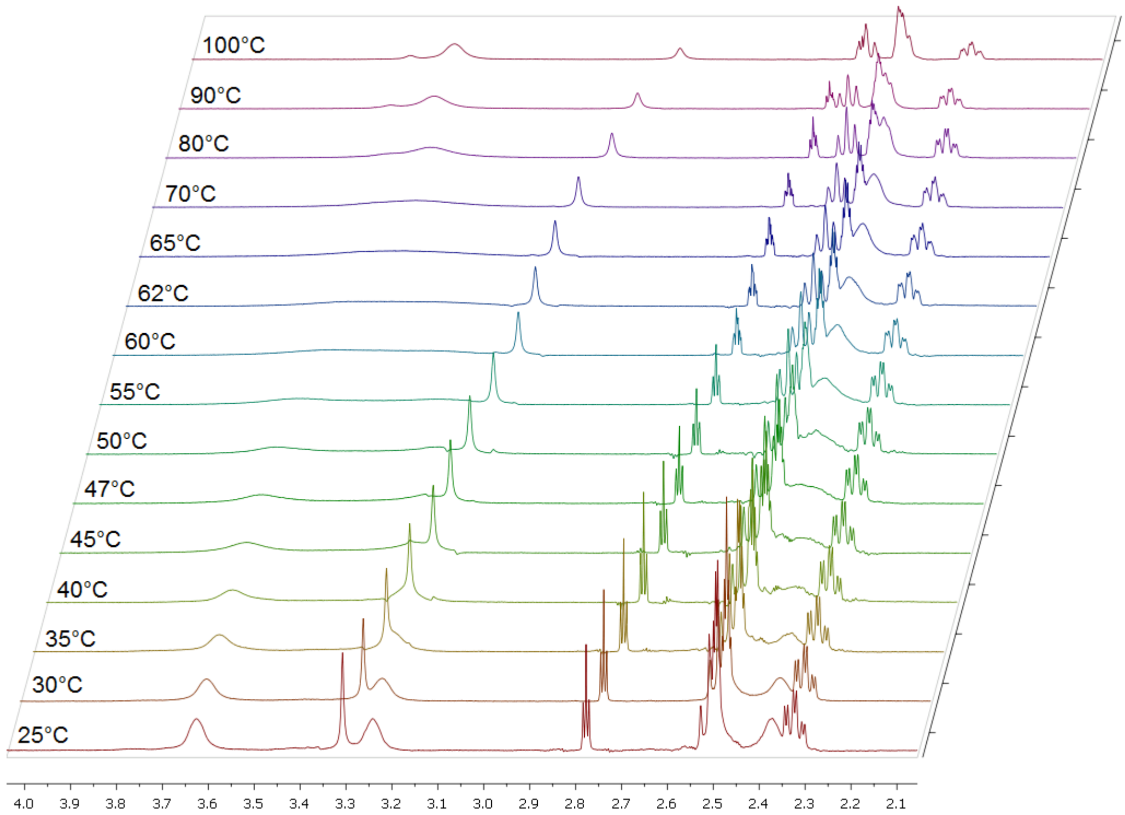

^{1}H NMR of **5a** measured at various temperatures (21–70 °C) in DMSO-d_6

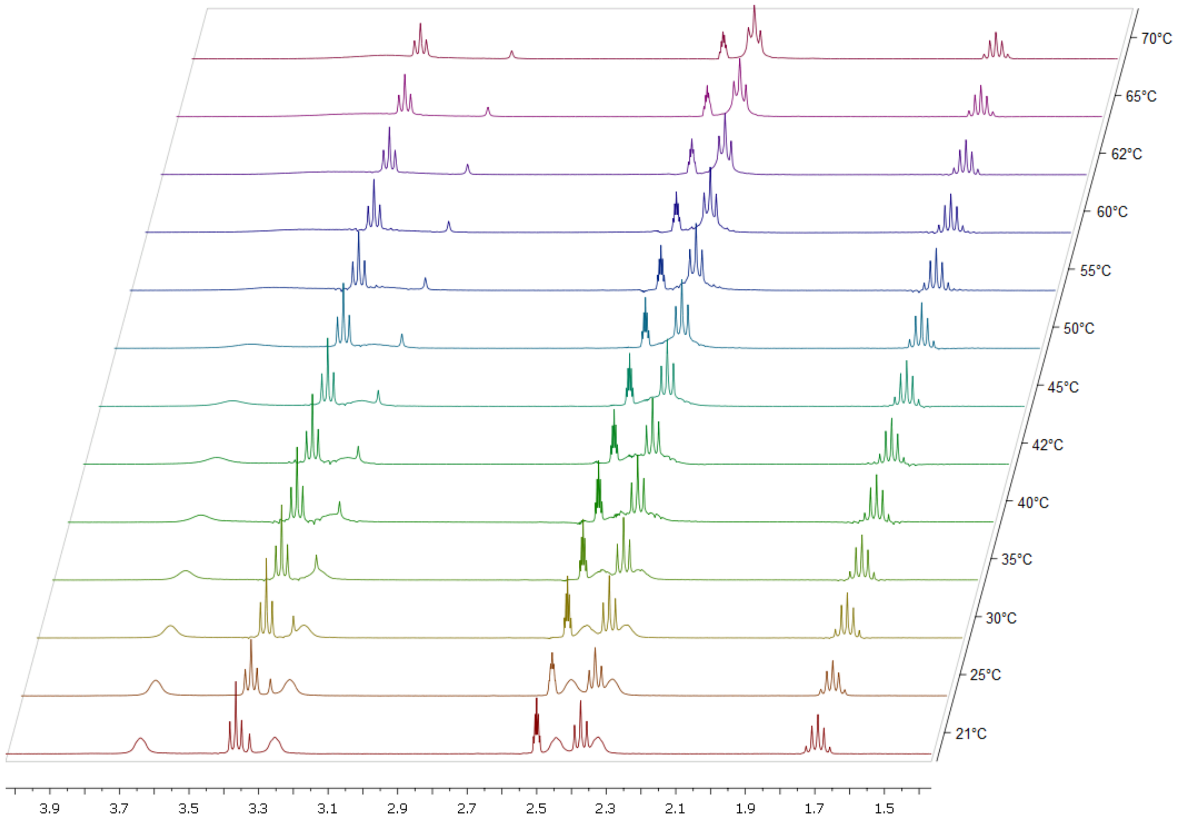

Radiochemical HPLC/TLC analyses

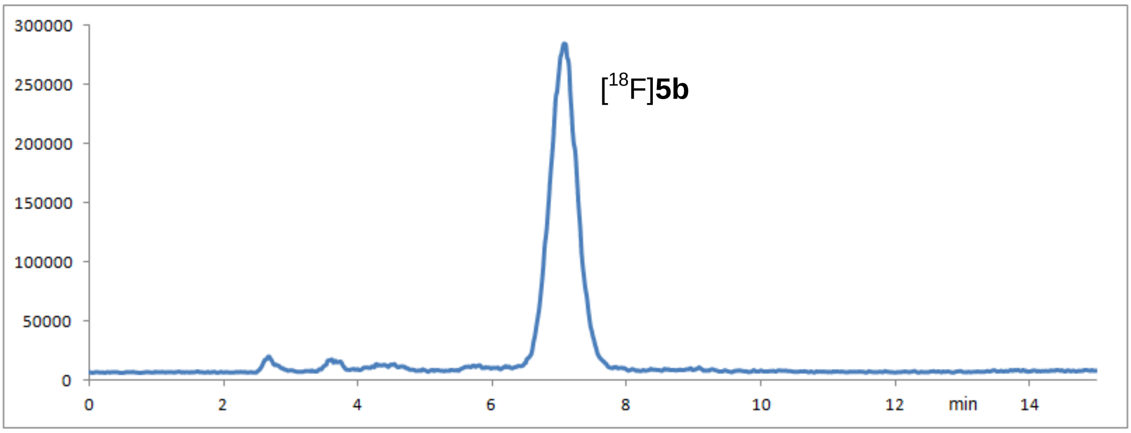

Radio-HPLC-chromatogram (γ-trace) of [^{18}F]**5b** (t_R = 7.1 min).

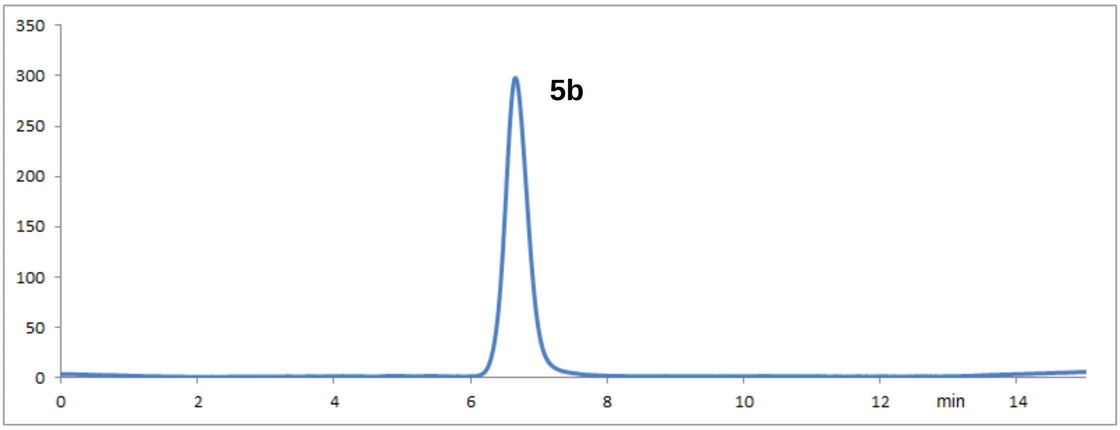

HPLC-chromatogram (UV-trace) of **5b** (t_R = 6.7 min).

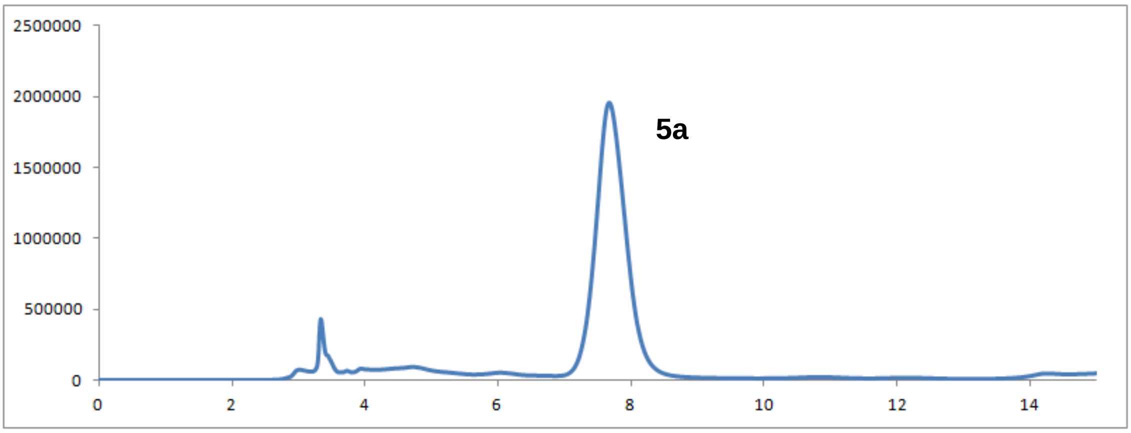

HPLC-chromatogram (UV-trace) of **5a** (t_R = 7.7 min).

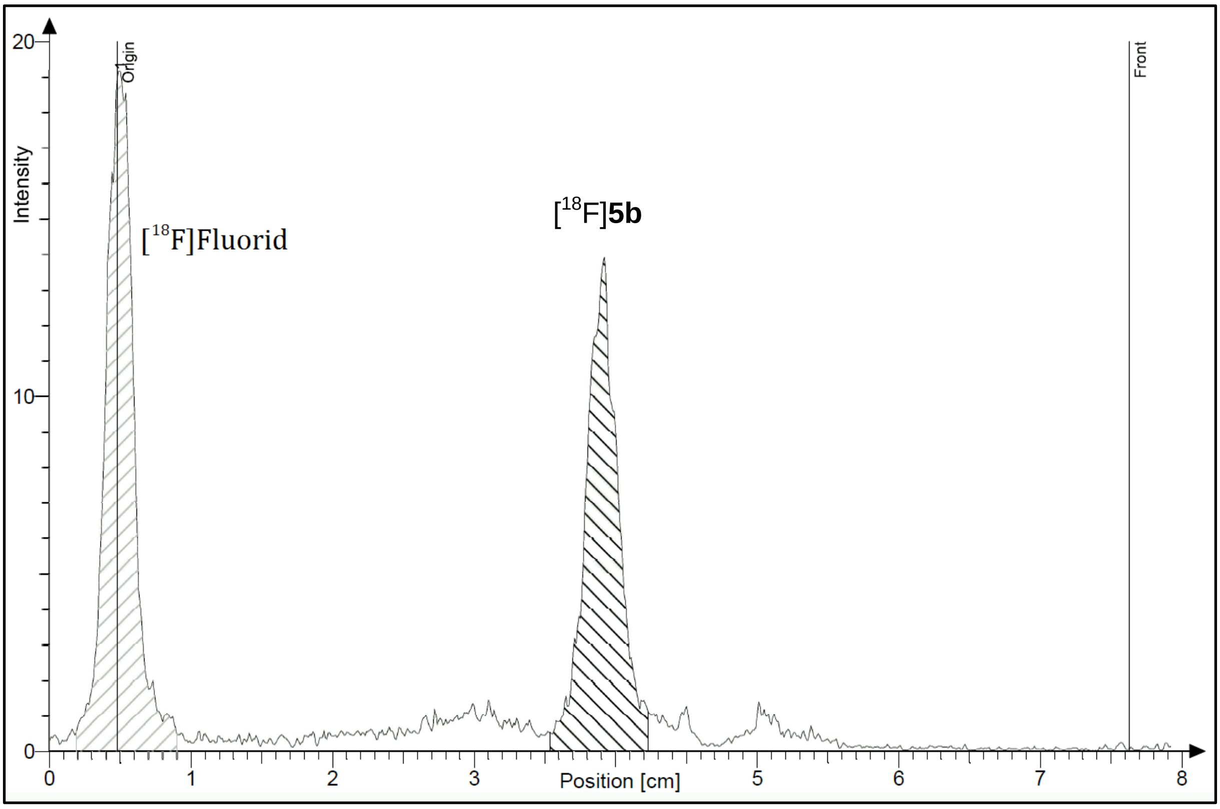

Radio-TLC (reaction mixture) of labeling of [^{18}F]**5b** R_f = 0.49 (ethanol).

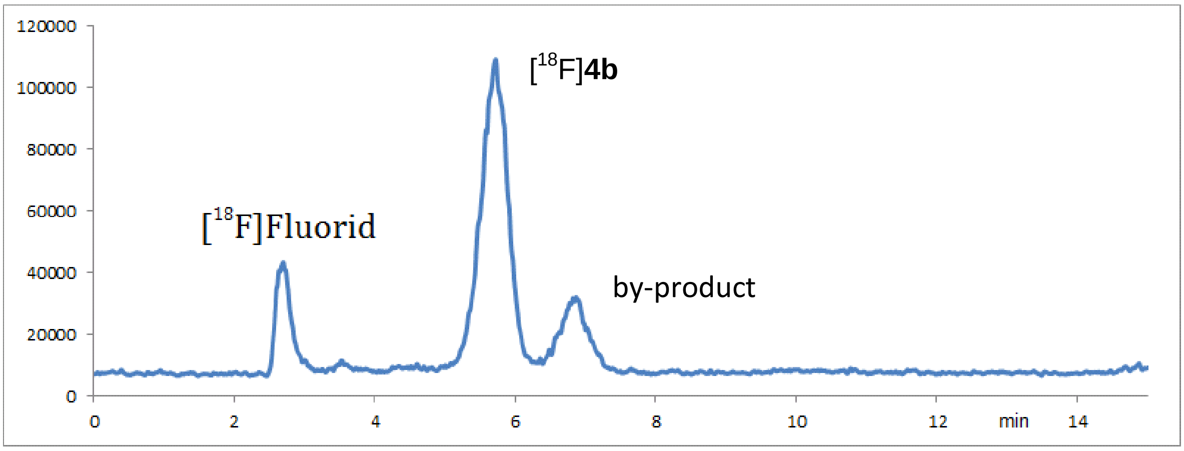

Radio-HPLC-chromatogram (γ-trace) of [^{18}F]**4b** (t_R = 5.7 min), the unknown byproduct (t_R = 6.9 min) and remaining [^{18}F]fluoride (t_R = 2.7 min).

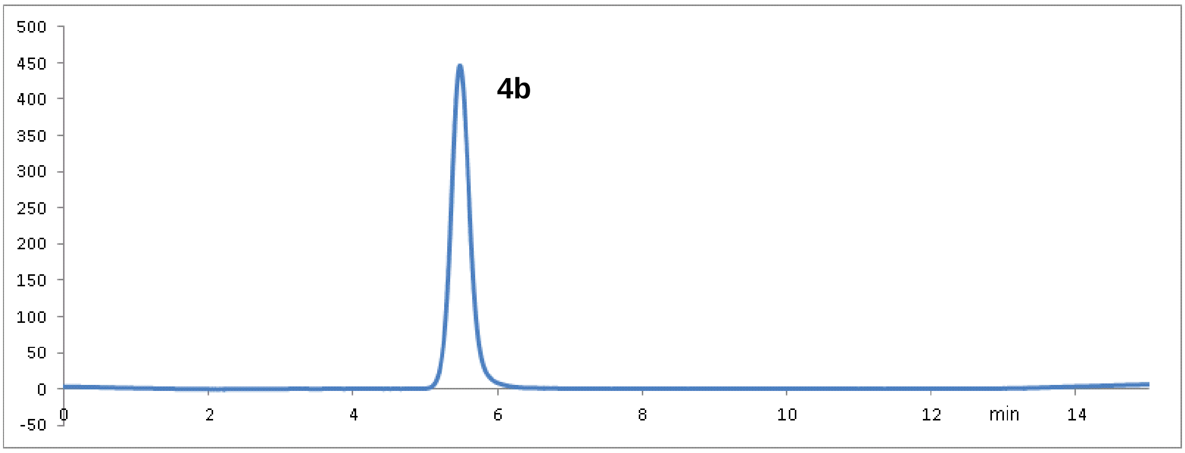

HPLC-chromatogram (UV-trace) of reference compound **4b** (t_R = 5.5 min).

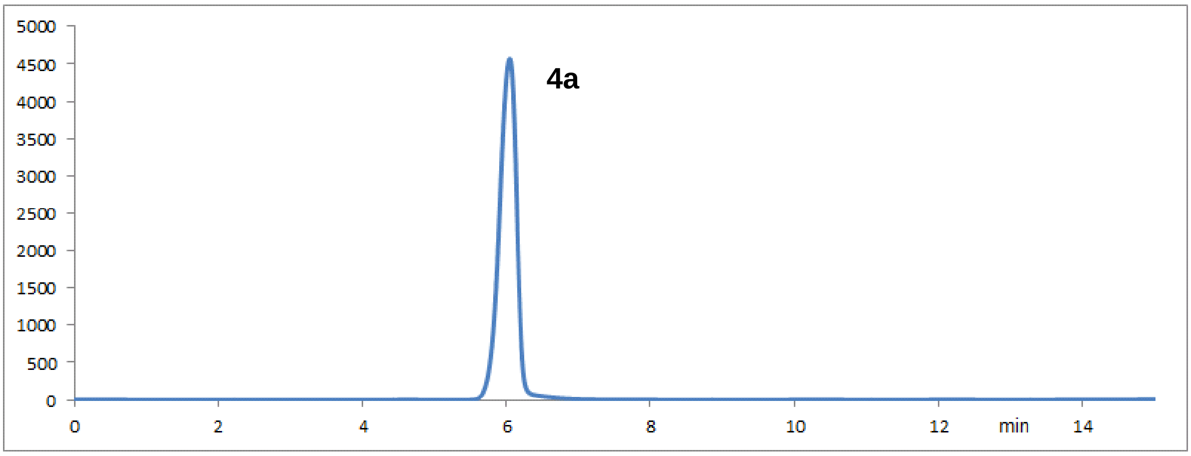

HPLC-chromatogram (UV-trace) of precursor **4a** (t_R = 6.0 min).*

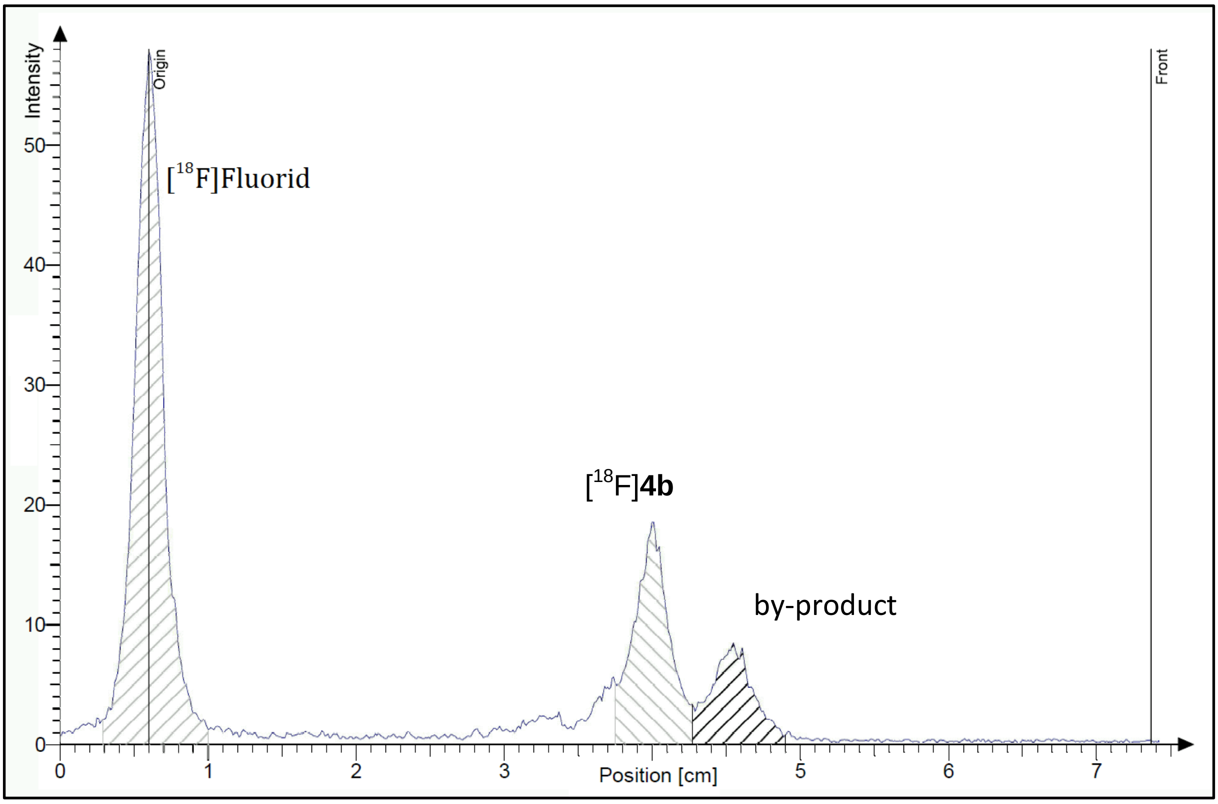

Radio-TLC (reaction mixture) of labeling of [^{18}F]**4b** (R_f = 0.50 in ethanol).

Chromatographic purifications of compounds 4a, 4b, 5a and 5b using Biotage

Biotage-chromatogram of compound **4a**

Biotage **Isolera Fraktionenbericht** 1.

Benutzer	mamat	Säulenposition	1
Probenname	cm514	Säule	SNAP 10g
Datum	2015-Nov-06 10.06	Racktyp	16x150 mm
Methode		Max. Fraktionenvol.	20 ml
Detektionsmodus	Manuell	Lösungsmittel A	Petrolether
UV1 (Überwachung)	254 nm	Lösungsmittel B	Ethyl acetate
UV2 (Überwachung)	280 nm		

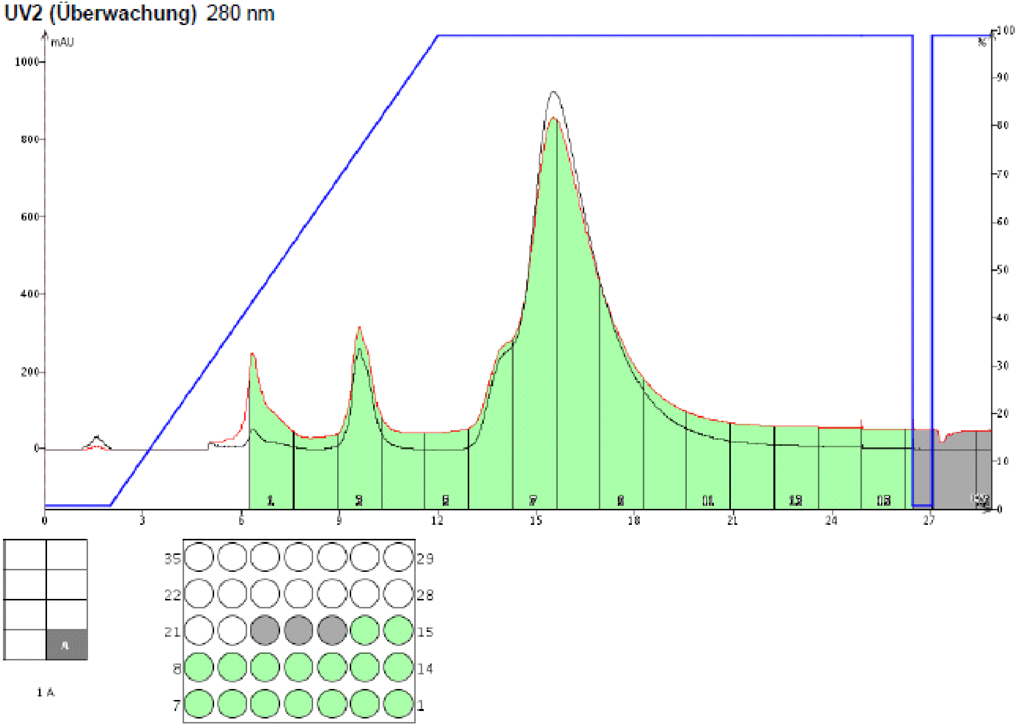

Biotage-chromatogram of **4b**

Biotage

Isolera Fraktionenbericht

1.

**Benutzer**	mamat	**Säulenposition**	1
**Probenname**	cm518 F	**Säule**	SNAP 10g
**Datum**	2015-Nov-19 11.09	**Racktyp**	16x150 mm
**Methode**	cm515	**Max. Fraktionenvol.**	20 ml
**Detektionsmodus**	Manuell	**Lösungsmittel A**	Petrolether
**UV1 (Überwachung)**	254 nm	**Lösungsmittel B**	Ethyl acetate
**UV2 (Überwachung)**	280 nm		

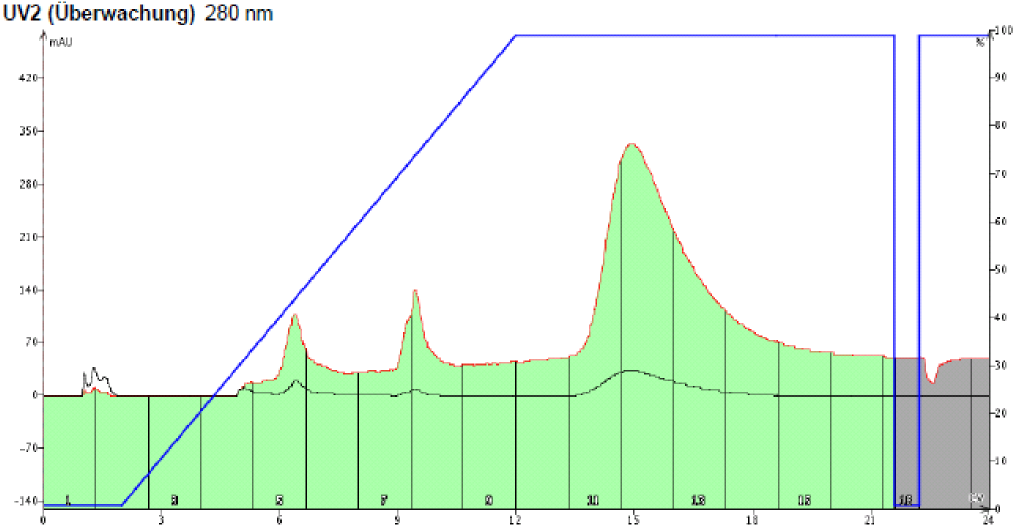

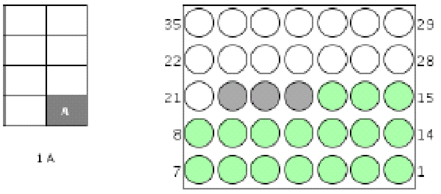

Biotage-chromatogram of **5a**

Biotage

Isolera Fraktionenbericht

1.

**Benutzer**	mamat	**Säulenposition**	1
**Probenname**	cm517 NO2	**Säule**	SNAP 10g
**Datum**	2015-Nov-19 09.24	**Racktyp**	16x150 mm
**Methode**	cm515	**Max. Fraktionenvol.**	20 ml
**Detektionsmodus**	Manuell	**Lösungsmittel A**	Petrolether
**UV1 (Überwachung)**	254 nm	**Lösungsmittel B**	Ethyl acetate
**UV2 (Überwachung)**	280 nm		

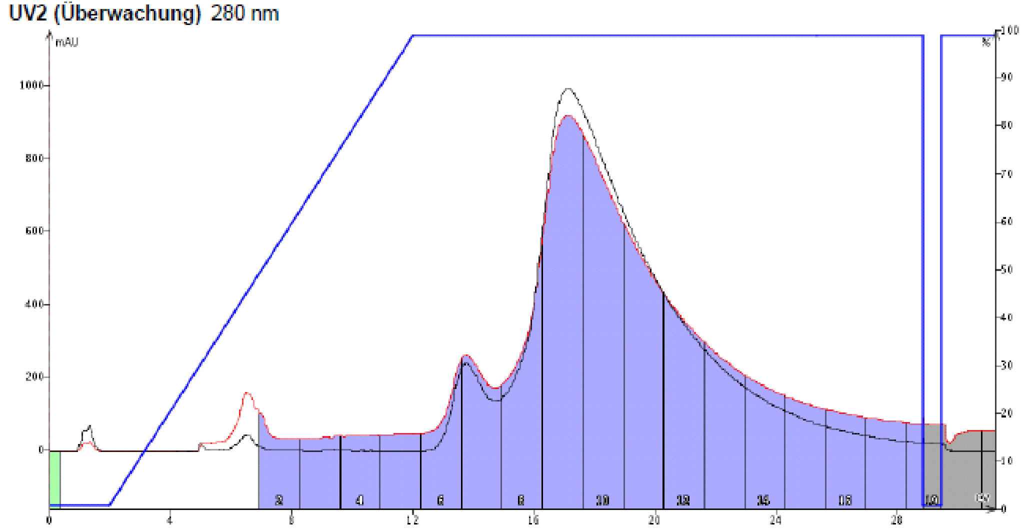

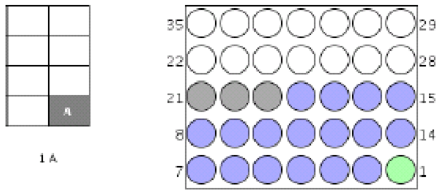

Biotage-chromatogram of **5b**

Biotage

Isolera Fraktionenbericht

1.

**Benutzer**	mamat	**Säulenposition**	1
**Probenname**	cm515	**Säule**	SNAP 10g
**Datum**	2015-Nov-12 10.18	**Racktyp**	16x150 mm
**Methode**	cm515	**Max. Fraktionenvol.**	20 ml
**Detektionsmodus**	Manuell	**Lösungsmittel A**	Petrolether
**UV1 (Überwachung)**	254 nm	**Lösungsmittel B**	Ethyl acetate
**UV2 (Überwachung)**	280 nm		

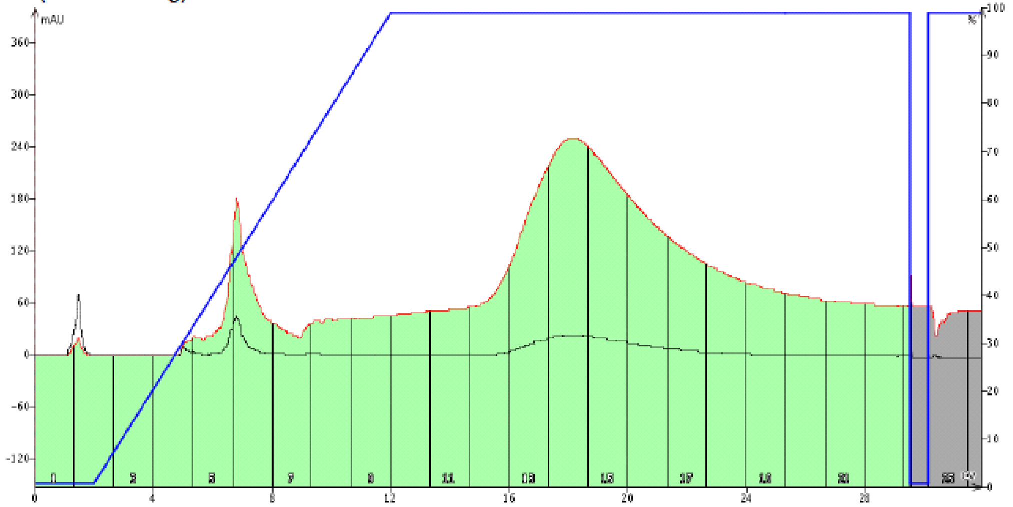

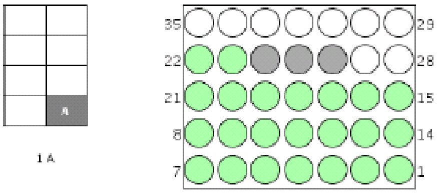

Supplement: File 1 — Copies of NMR spectra of investigated piperazines, radioHPLC chromatograms, and separation methods for piperazines. [file Beilstein_J_Org_Chem-12-2478-s001.pdf]
